# Type I interferon sensing unlocks dormant adipocyte inflammatory potential

**DOI:** 10.1038/s41467-020-16571-4

**Published:** 2020-06-02

**Authors:** Calvin C. Chan, Michelle S. M. A. Damen, Maria E. Moreno-Fernandez, Traci E. Stankiewicz, Monica Cappelletti, Pablo C. Alarcon, Jarren R. Oates, Jessica R. Doll, Rajib Mukherjee, Xiaoting Chen, Rebekah Karns, Matthew T. Weirauch, Michael A. Helmrath, Thomas H. Inge, Senad Divanovic

**Affiliations:** 1 Medical Scientist Training Program, Cincinnati Children’s Hospital Medical Center and the University of Cincinnati College of Medicine, Cincinnati, OH 45220 USA; 2 Immunology Graduate Program, Cincinnati Children’s Hospital Medical Center and the University of Cincinnati College of Medicine, Cincinnati, OH 45220 USA; 3 Department of Pediatrics, University of Cincinnati College of Medicine, Cincinnati, OH 45220 USA; 4 Division of Immunobiology, Cincinnati Children’s Hospital Medical Center, Cincinnati, OH 45229 USA; 5 The Center for Autoimmune Genomics and Etiology, Cincinnati Children’s Hospital Medical Center, Cincinnati, OH 45229 USA; 6 Division of Gastroenterology, Hepatology and Nutrition, Cincinnati Children’s Hospital Medical Center, Cincinnati, OH 45229 USA; 7 Division of Biomedical Informatics, Cincinnati Children’s Hospital Medical Center, Cincinnati, OH 45229 USA; 8 Divsion of Developmental Biology, Cincinnati Children’s Hospital Medical Center, Cincinnati, OH 45229 USA; 9 Division of Pediatric General and Thoracic Surgery, Cincinnati Children’s Hospital Medical Center, Cincinnati, OH 45229 USA; 10 Center for Stem Cell & Organoid Medicine, Cincinnati Children’s Hospital Medical Center, Cincinnati, OH 45229 USA; 11 Department of Surgery, Children’s Hospital Colorado, Aurora, CO 80045 USA; 12 Center for Inflammation and Tolerance, Cincinnati Children’s Hospital Medical Center, Cincinnati, OH 45229 USA; 13 Present Address: Divisions of Neonatology and Developmental Biology, David Geffen School of Medicine at UCLA, Mattel Children’s Hospital UCLA, Los Angeles, CA USA

**Keywords:** Interferons, Inflammation, Obesity

## Abstract

White adipose tissue inflammation, in part via myeloid cell contribution, is central to obesity pathogenesis. Mechanisms regulating adipocyte inflammatory potential and consequent impact of such inflammation in disease pathogenesis remain poorly defined. We show that activation of the type I interferon (IFN)/IFNα receptor (IFNAR) axis amplifies adipocyte inflammatory vigor and uncovers dormant gene expression patterns resembling inflammatory myeloid cells. IFNβ-sensing promotes adipocyte glycolysis, while glycolysis inhibition impeded IFNβ-driven intra-adipocyte inflammation. Obesity-driven induction of the type I IFN axis and activation of adipocyte IFNAR signaling contributes to obesity-associated pathogenesis in mice. Notably, IFNβ effects are conserved in human adipocytes and detection of the type I IFN/IFNAR axis-associated signatures positively correlates with obesity-driven metabolic derangements in humans. Collectively, our findings reveal a capacity for the type I IFN/IFNAR axis to regulate unifying inflammatory features in both myeloid cells and adipocytes and hint at an underappreciated contribution of adipocyte inflammation in disease pathogenesis.

## Abstract (web summary)

White adipose inflammation can occur in obesity and is at least in part mediated by inflammatory immune cells. Here the authors show that the Type I Interferon/Interferon alpha-beta receptor axis promotes an inflammatory, glycolysis associated adipocyte phenotype.

## Introduction

Obesity is an unabated public health problem of the first order^1,2^. The pathological expansion of adipocytes in obesity creates a highly dynamic and multicellular inflammatory milieu within the white adipose tissue (WAT)^3^. Such low-grade chronic inflammation propagates the pathogenesis of obesity-associated sequelae including, type 2 diabetes mellitus (T2D) and non-alcoholic fatty liver disease (NAFLD)^4^. In contrast to myeloid cells^5^, regulation of adipocyte inflammatory potential and its contribution to obesity-associated inflammation is poorly understood. Like myeloid cells, adipocytes produce proinflammatory cytokines^6^, express innate immune receptors (e.g. Toll-like receptor (TLR))^7^ and major histocompatibility complex (MHC) class I and class II molecules and present antigens^8,9^. However, whether adipocytes possess intrinsic potential to behave “like” myeloid cells and what mechanisms regulate adipocyte inflammatory potential remains poorly defined.

Type I Interferons (IFN), predominantly IFNα and IFNβ, are dynamic immune mediators that orchestrate both innate and adaptive immune responses, including fine tuning of myeloid cell inflammatory vigor^10^. IFNβ is expressed ubiquitously, while hematopoietic cells are the primary source of IFNα. Obesity-associated metabolic endotoxemia (lipopolysaccharide; LPS)^11^ activates TLR signaling cascades and TLR signaling is a robust inducer of type I IFN production. Type I IFN engagement of ubiquitously expressed heterodimeric transmembrane receptor IFNα receptor (IFNAR) initiates various inflammatory signaling hubs^12^.

Existing literature on the contribution of the IFNAR axis to obesity-associated sequelae suggests either detrimental^13–18^ or beneficial^19,20^ effects. However, to our knowledge, the contribution of the type I IFN/IFNAR axis in regulation of adipocyte inflammatory potential has not been investigated. Given the relevance of type I IFN/IFNAR axis in regulation of myeloid cell inflammatory potential, and partial commonality in inflammatory functions between myeloid cells and adipocytes, we hypothesized that the activation of the type I IFN/IFNAR axis in adipocytes would uncover immune-like inflammatory signatures and exacerbate adipocyte-intrinsic inflammatory vigor.

Here we demonstrate that the type I IFN/IFNα receptor (IFNAR) axis uncovers glycolysis-associated adipocyte inflammatory vigor and dormant gene expression patterns that resemble inflammatory myeloid cells. Obesity induces the type I IFN axis and adipocyte IFNAR signaling contributes to obesity-associated disease pathogenesis in mice. The type I IFN/IFNAR axis-associated signatures positively correlate with obesity-driven metabolic derangements in humans and IFNβ effects are conserved in human adipocytes. Combined our observations reveal potential for the type I IFN/IFNAR axis to unify inflammatory characteristics in both myeloid cells and adipocytes.

## Results

### Type I IFN/IFNAR axis augments adipocyte inflammatory potential

**Fig. 1:**
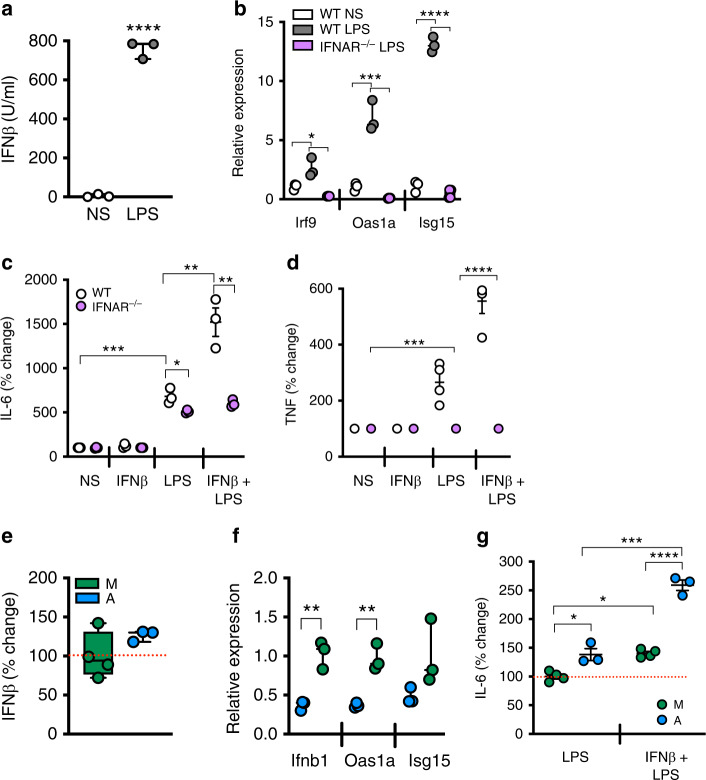
IFNβ/IFNAR axis exacerbates adipocyte immune potential. Primary adipocytes or bone-marrow-derived macrophages isolated from chow-diet-fed WT and IFNAR^−/−^ mice were treated with saline (NS), IFNβ (250 U/ml) or LPS (100 ng/ml) as indicated. **a** Quantified IFNβ protein levels in adipocyte culture supernatants by type I IFN activity assay. **b** mRNA expression by qPCR of indicated type I IFN axis genes in adipocytes, relative expression to WT NS. **c** IL-6 and **d** TNF protein levels in adipocyte culture supernatants quantified by ELISA; % change over NS. **e** Quantified IFNβ protein levels in adipocytes and macrophage culture supernatants by type I IFN activity assay; % change to macrophage. **f** mRNA expression of indicated type I IFN axis genes by qPCR in adipocytes and macrophage, relative expression to macrophage. **g** IL-6 protein levels in stimulated macrophages and adipocytes under indicated conditions quantified by ELISA; % change to LPS-stimulated macrophages. **a**−**d** Representative of three independent experiments, *n* = 3−4/condition. **e**−**g** Representative of three independent experiments, *n* = 3−4/condition. For bar graphs, data represents mean ± SEM. **a**−**g** For box plots, the midline represents the mean, boxes represent the interquartile range and whiskers show the full range of values. **a**−**d** Unpaired two-tailed Student’s *t* test. *P < 0.05, **P < 0.01, ***P < 0.001, ****P < 0.0001. **e**−**g** Unpaired two-tailed Student’s *t* test. *P < 0.05, **P < 0.01, ***P < 0.001, ****P < 0.0001. M denotes macrophage and A denotes adipocyte. Source data are provided as a Source data file.

Obesity is linked with augmented levels of various systemic TLR triggers (e.g. LPS^11^, DNA^21^) known to induce type I IFN production. Type I IFN engagement of the ubiquitously expressed IFNAR initiates activation of inflammatory mediators including STAT3, interleukin-6 (IL-6), tumor necrosis factor (TNF) and various chemokines^12^. Whether LPS stimulation is sufficient to activate the type I IFN axis in primary adipocytes, as it does in myeloid cells, is underdefined. LPS treatment of mouse primary adipocytes induced IFNβ production (Fig. 1a) and mRNA expression of type I IFN signature genes including *Irf9*, *Oas1a* and *Isg15* in an IFNAR-dependent manner (Fig. 1b). Further, as in myeloid cells^10,22^, IFNβ treatment enhanced adipocyte IFNAR-dependent, LPS-driven proinflammatory cytokine production (Fig. 1c, d). Levels of LPS-driven IFNβ production (Fig. 1e), LPS-driven mRNA expression of the type I IFN signature genes (Fig. 1f) and IFNβ + LPS-driven inflammatory vigor (Fig. 1g) in adipocytes mirrored that observed in myeloid cells. Priming of adipocytes was not restricted to IFNβ, as an IFNα subtype (e.g. IFNα4) similarly enhanced LPS-driven IL-6 production (Supplementary Fig. 1a). In addition to *Tlr4*, primary adipocytes also expressed *Tlr2*, *Tlr3*, and *Tlr9* (Supplementary Fig. 1b) and activation of TLR2 (Pam2Cys) or TLR3 (Poly I:C) signaling in adipocytes was sufficient to induce IL-6 and IFNβ production and activate the type I IFN axis (Supplementary Fig. 1c−f). Overall these findings suggest that akin to myeloid cells, various TLR ligands can potently induce proinflammatory cytokine production and activate the type I IFN axis in adipocytes. In addition, our data indicate that activation of the type I IFN/IFNAR axis regulates adipocyte inflammatory vigor.

### Type I IFN uncovers dormant adipocyte inflammatory networks

**Fig. 2:**
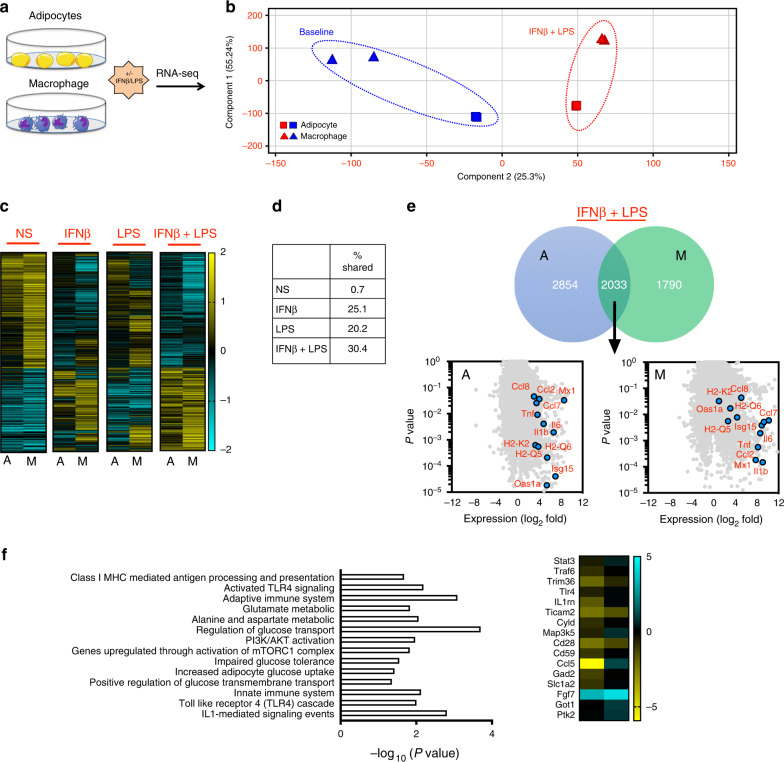
Type I IFN/IFNAR axis drives convergence between adipocyte and macrophage inflammatory gene signature. Adipocytes and macrophages isolated from chow-diet-fed WT mice were treated with (NS), IFNβ (250 U/ml) and/or LPS (100 ng/ml) and subjected to RNA-seq analysis. **a** Schematic overview. **b** Principal component analysis of RNA-seq analysis, distance of component 2 (*X*-axis) delineates the similarity of gene expression patterns between cell types. **c** Heat maps of RNA-seq analyses in adipocytes and macrophages treated under indicated conditions. **d** Percent of shared genes between adipocytes and macrophages treated under indicated conditions. **e** Venn diagram (top) and volcano plots (bottom) of genes differentially expressed under IFNβ + LPS treatment in adipocytes and macrophages. **f** Adipocyte ontology pathways and heat map of representative genes distinct to IFNβ + LPS treatment. **a**−**f** A single experiment, *n* = 2/condition. A denotes adipocytes. M denotes macrophages. Source data are provided as a Source data file.

This similar type I IFN mediated tuning of inflammatory vigor between adipocytes and myeloid cells (e.g. macrophages) prompted us to investigate the breadth of their shared functionality. Utilizing an unbiased RNA-seq approach (Fig. 2a), principal component analysis revealed that despite clear distinction at baseline between adipocytes and bone-marrow-derived macrophages, treatment with IFNβ followed by LPS drove adipocytes and macrophages to converge towards a similar gene expression signature (Fig. 2b, c). At baseline, only 35 out of the 2500 most highly expressed genes (0.7%; most overlapped genes are putative) in adipocytes and macrophages were shared, indicating a comparison of distinct cell types (Fig. 2d; Supplementary Fig. 2a). Of note, while IFNβ or LPS alone increased the overlap of gene expression patterns (1206 [25.1%], 758 [20.2%] respectively) between the cell types (Fig. 2d; Supplementary Fig. 2b), IFNβ + LPS combination treatment led to the greatest convergence of regulated genes (2033 [30.4%]; Fig. 2d). Comparably upregulated genes between the adipocytes and macrophages, after combined IFNβ + LPS treatment, included those associated with broad activation of inflammatory cascades and antigen presentation (Fig. 2e; Supplementary Fig. 2c, d). Complementary computational analyses of enriched transcription factor (TF) binding site “motifs” and ChIP-seq and DNase-seq peaks highlighted a convergence between IFNβ + LPS-stimulated adipocytes and macrophages (e.g. STATs, IRFs, NF-κb, chromatin accessibility; Supplementary Fig. 2e, f). Notably, in adipocytes, 764 [42%] genes were overlapped between IFNβ + LPS and IFNβ-alone treatment, while only 151 [8.3%] genes were overlapped between IFNβ + LPS and LPS-alone treatment (Supplementary Fig. 3a). Closer examination of pathways significantly augmented by combined IFNβ + LPS treatment (>2-fold over IFNβ or LPS alone) in adipocytes revealed ontologies associated with diabetic complications, TNF and IL-6 signaling, TLR signaling, and secreted soluble factors (e.g. IL-15, CXCL9, CXCL10, CCL5; Supplementary Fig. 3b). Additionally, inhibition of Jak1 abrogated IFNβ augmentation of adipocyte inflammatory vigor and activation of the IFNAR axis (Supplementary Fig. 3c, d). Pathways that were distinct to IFNβ + LPS treatment in adipocytes included class I MHC processing and presentation, adaptive and innate immune system, PI3K/AKT activation, impaired glucose tolerance, increased adipocyte glucose uptake, and regulation of glucose transmembrane transport (Fig. 2f). Collectively these findings suggest that adipocytes possess a dormant underlying immunological capacity similar to bone-marrow-derived cells of myeloid origin and that the activation of the type I IFN/IFNAR axis in adipocytes is, in part, responsible for uncovering adipocyte inflammatory clades.

### Type I IFN/IFNAR axis promotes adipocyte glycolysis

**Fig. 3:**
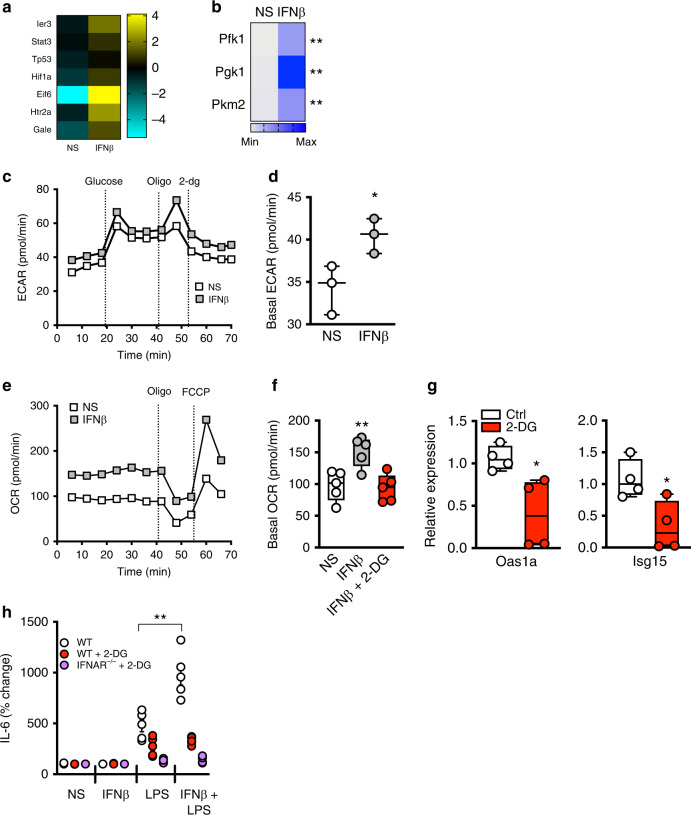
IFNβ modifies glycolysis-associated inflammatory vigor in adipocytes. **a** Differential expression of representative genes in pathways associated with regulation of glycolysis in adipocytes from RNA-seq analysis. **b** WT adipocytes were treated in the presence or absence of IFNβ (250 U/ml) and mRNA expression of indicated glycolytic pathway-associated genes measured by qPCR, relative expression compared to NS. **c**−**f** Adipocyte cellular bioenergetics as determined by Seahorse XF96 analyzer with sequential injection of glucose (2 mM), oligomycin (2 µg/ml), 2-Deoxy-d-glucose (2-DG, 10 mM), or FCCP [1 mM] as indicated. **c** Mean extracellular acidification rate (ECAR). **d** Basal ECAR. **e** Mean oxygen consumption rate (OCR). **f** Basal OCR in the presence or absence of pretreatment with 2-DG (2 mM). **g** IFNβ (250 U/ml)-treated adipocyte expression of indicated type I IFN axis genes measured by qPCR in the presence or absence of 2-DG (2 mM), relative expression to ctrl. **h** IL-6 protein levels in adipocyte supernatant quantified by ELISA; % change over NS. **a** A single experiment, *n* = 2/condition. **b** Representative of three independent experiments, *n* = 3/condition. **c**−**f** Representative of three independent experiments, *n* = 3−5/condition. **g** Representative of three independent experiments, *n* = 3−4/condition. **h** Data combined from two independent experiments, *n* = 3−5/condition. **c**−**h** For box plots, the midline represents the mean, boxes represent the interquartile range and whiskers show the full range of values. In line graphs data represent mean ± SEM. **b**, **d**, **f**−**h** Unpaired two-tailed Student’s *t* test. *P < 0.05, **P < 0.01. Source data are provided as a Source data file.

We next sought to determine the means by which type I IFN/IFNAR axis uncovers adipocyte inflammatory potential. Alteration of glycolysis and fatty acid oxidation regulates myeloid cell inflammatory capacity^23,24^, and glucose metabolism mediates IFNβ antiviral capacity^25^. Our unbiased RNA-seq analysis of IFNβ-treated adipocytes also revealed augmented expression of genes regulating glycolytic pathways (e.g. *Hif1a*^26^, *Eif6*^27^; Fig. 3a). To examine the direct effect of IFNβ on adipocyte metabolic flux through glycolysis, the expression of key glycolysis-associated enzymes, lactate production, and cellular respiration were quantified. IFNβ treatment of adipocytes enhanced phosphofructokinase 1 (*Pfk1*), phosphoglycerate kinase (*Pgk1*) and pyruvate kinase (*Pkm2*) mRNA expression (Fig. 3b), promoted basal extracellular acidification rate (ECAR) (Fig. 3c, d), without altering lactate production (Supplementary Fig. 4a), and increased cellular respiration (Oxygen Consumption Rate [OCR]) (Fig. 3e, f). Combined IFNβ + LPS treatment did not further enhance ECAR (Supplementary Fig. 4b). These data hinted that IFNβ could alter aerobic glycolysis in adipocytes. To begin to delineate metabolic pathways modulated by IFNβ and to examine if such alterations are linked to glycolysis, adipocytes were treated with IFNβ in the presence of inhibitors of fatty acid oxidation (etomoxir) or glycolysis (2-Deoxy-d-Glucose (2-DG)). Only 2-DG, but not etomoxir, treatment reversed the IFNβ-mediated increase in basal cellular respiration (Fig. 3f; Supplementary Fig. 4c, d), dampened the expression of IFNβ-driven signature genes (*Oas1a* and *Isg15*; Fig. 3g) and depressed IFNβ-driven augmentation of adipocyte inflammatory potential (Fig. 3h; Supplementary Fig. 4e). Combined, these findings suggest that the type I IFN axis alters adipocyte core metabolism, possibly through aerobic glycolysis, and that such modification is associated with the enhancement of adipocyte inflammatory potential.

### IFNAR activation exacerbates obesity-associated sequelae

**Fig. 4:**
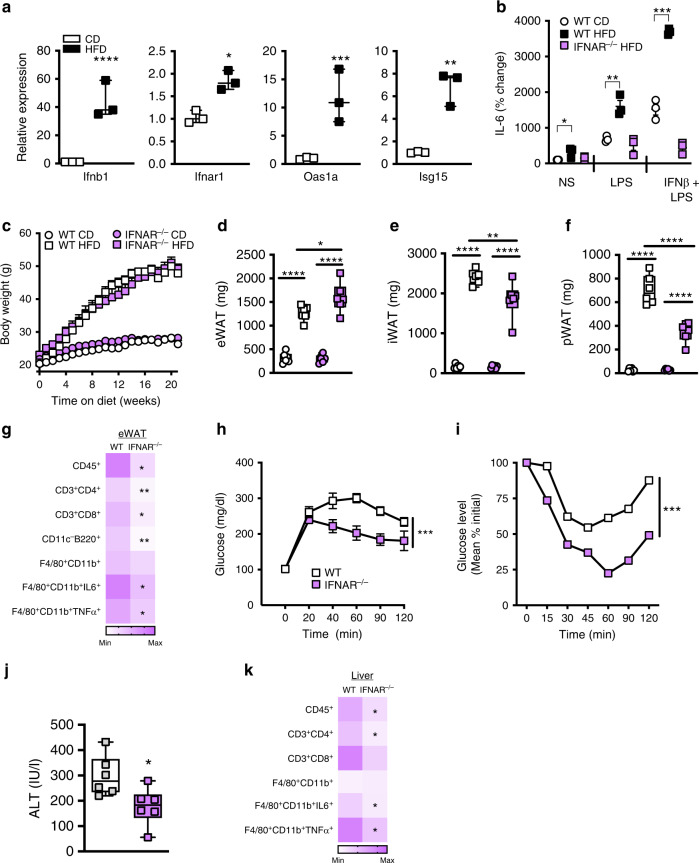
Type I IFN/IFNAR axis contributes to the pathogenesis of obesity-associated sequelae. **a**, **b** Adipocytes were isolated from WT mice placed on a high-fat diet (HFD) or low-fat chow diet (CD) for 8 weeks. **a** mRNA expression of the indicated type I IFN axis genes by qPCR in primary adipocytes, relative expression to CD. **b** Primary adipocytes treated with saline (NS), IFNβ (250 U/ml) or LPS (100 ng/ml) as indicated and IL-6 protein levels in supernatant were quantified by ELISA; % change over NS. **c**−**k** WT and IFNAR^−/−^ mice were fed HFD for 22 weeks. **c** Total body weight over time. **d** eWAT, **e** iWAT, **f** pWAT weights. **g** Average eWAT immune cell infiltration measured by flow cytometry. **h** Glucose and **i** insulin tolerance tests. **j** Systemic alanine transaminase (ALT) quantified at time of harvest. **k** Average liver immune cell infiltration measured by flow cytometry. **a**, **b** Representative of two independent experiments, *n* = 3/condition. **c**−**k** Representative of three independent experiments, *n* = 6−7/condition. **a**, **b**, **d**−**f**, **j** For box plots, the midline represents the mean, boxes represent the interquartile range and whiskers show the full range of values. **c**, **g**, **h**−**I**, **k** For line graphs and heat maps data represent mean ± SEM. **a**, **b**, **d**−**g**, **j**, **k** Unpaired two-tailed Student’s *t* test. *P < 0.05, **P < 0.01, ***P < 0.001, ****P < 0.0001. **c**, **h**, **i** Area under curve. ***P < 0.001. Source data are provided as a Source data file.

Adipocytes are important players in obesity development and obesity is associated with activation of various inflammatory processes^28^. WT mice fed a high-fat diet (HFD), compared to chow diet (CD), had an enhanced expression of type I IFN signature genes *Oas1a* and *Isg15* in spleen, liver, and various fat depots (iWAT, eWAT, pWAT) (Supplementary Fig. 5). As adipocytes comprise the core of WAT, expression and activation of type I IFN axis in adipocytes was examined next. Primary adipocytes from HFD-fed WT mice, compared to CD-fed controls, displayed an augmented type I IFN signature including *Ifnb1*, *Ifnar1*, *Oas1a*, and *Isg15* (Fig. 4a). Further, in an IFNAR-dependent manner, IFNβ primed adipocytes from HFD mice, compared to CD-fed controls, were significantly more vigorous in their IL-6 output after LPS challenge (Fig. 4b).

Pathological accumulation of WAT/adipocytes and inflammation are hallmarks of obesity development and pathogenesis of obesity-associated sequelae. IFNAR signaling modulates inflammatory potential of both immune cells and adipocytes. HFD-fed total body IFNAR^−/−^ mice and WT mice exhibited similar obesity as assessed by lack of differential total body weight (Fig. 4c; in agreement with a recent report^13^), energy expenditure, food intake, systemic cholesterol, total body adiposity, BAT *Ucp-1* expression and WAT gross morphological appearance (Supplementary Fig. 6a−f). However, genetic modulation of IFNAR signaling altered WAT distribution (i.e. increased eWAT mass (Fig. 4d), decreased inguinal (Fig. 4e) and perirenal WAT mass (Fig. 4f)). Progressive decline in eWAT is associated with increased adipocyte death, enhanced inflammation and total body insulin resistance^29^. Despite increased eWAT size, lack of IFNAR signaling correlated with reduced eWAT total immune cell infiltration (CD45^+^) and specifically with diminished numbers of infiltrating T cells (CD3^+^CD4^+^ and CD3^+^CD8^+^) and B cells (CD11c^−^B220^+^) (Fig. 4g; Supplementary Fig. 7; Supplementary Table 1) and expression of the T- and B-cell chemoattractants (T cells: *Cxcl10, Cxcl9;* B cells: *Cxcl13* and *Ltb4r*) (Supplementary Fig. 8). Although the total number of eWAT infiltrating macrophages (F4/80^+^CD11b^+^) was similar between WT and IFNAR^−/−^ mice, lack of IFNAR signaling reduced percent of macrophages producing proinflammatory cytokines central to obesity pathogenesis^5^ (IL-6 and TNF; Fig. 4g; Supplementary Fig. 7; Supplementary Table 1).

In agreement with reduced inflammatory setting, compared to their age-, diet- and weight-matched WT controls, obese total body IFNAR^−/−^ mice exhibited improved glucose metabolism, as determined by glucose and insulin tolerance tests (Fig. 4h−i). Co-housing did not alter weight gain or protection from glucose dysmetabolism in IFNAR^−/−^ mice (Supplementary Fig. 9). Further, despite similar hepatic steatosis (cholesterol and triglyceride levels) and liver morphology between obese IFNAR^−/−^ and WT counterparts (Supplementary Fig. 10a, b), obese IFNAR^−/−^ mice had attenuated hepatocellular injury as measured by systemic alanine transaminase (ALT) levels (Fig. 4j; Supplementary Fig. 10c). Reduced hepatocellular damage in obese IFNAR^−/−^ mice correlated with decreased total hepatic immune cell numbers and numbers of CD4^+^ T cells, but similar numbers of liver infiltrating CD8^+^ T cells and macrophages (Fig. 4k; Supplementary Table 2). However, as in eWAT, total body deletion of IFNAR signaling reduced percent of liver macrophages producing proinflammatory cytokines (IL-6 and TNF) (Fig. 4k; Supplementary Table 2). These findings suggest that obesity promotes activation of the type I IFN/IFNAR axis and that type I IFN sensing by IFNAR modulates inflammation and pathogenesis of obesity-associated sequelae independent of weight gain.

### Adipocyte-intrinsic IFNAR contributes to obesity-associated sequelae

**Fig. 5:**
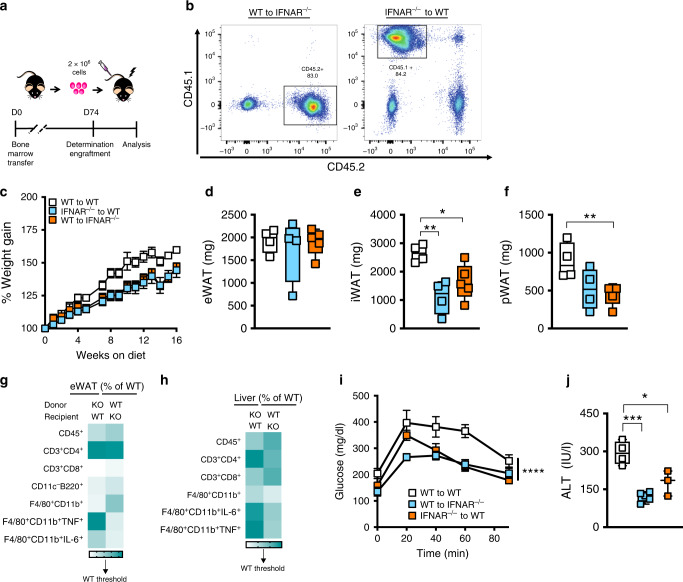
Hematopoietic and nonhematopoietic IFNAR expression contribute to the pathogenesis of obesity-associated sequelae. Reciprocal bone marrow transfers (BMT) between WT (CD45.2) and IFNAR^−/−^ (CD45.1) mice were performed and successful reconstitution was confirmed at d74 post-transfer by flow cytometry. Reconstituted mice were place on HFD for 18 weeks. **a** Schematic diagram of reciprocal BMT. **b** Reconstitution analysis by flow cytometry. **c** % weight gain over time. **d** eWAT, **e** iWAT, **f** pWAT weights. **g** Average eWAT immune cell infiltration measured by flow cytometry; % change over WT. **h** Average liver immune cell infiltration measured by flow cytometry; % change over WT. **i** Glucose tolerance test. **j** Systemic ALT quantified at time of harvest. **a**−**j** A single experiment, *n* = 4−5/condition. **c**, **g**−**i** For line graphs and heat maps data represent mean ± SEM. **d**−**f**, **j** For box plots, the midline represents the mean, boxes represent the interquartile range and whiskers show the full range of values. **d**−**h**, **j** Unpaired two-tailed Student’s *t* test. *P < 0.05, **P < 0.01, ***P < 0.001. **c**, **i** Area under the curve. ****P < 0.0001. Source data are provided as a Source data file.

The contribution of hematopoietic or nonhematopoietic IFNAR expression to obesity-associated metabolic sequelae, via reciprocal bone marrow transfers between WT and IFNAR^−/−^ mice, was examined next (Fig. 5a). Successful reconstitution was confirmed by flow cytometry (Fig. 5b). Both hematopoietic (KO to WT) and nonhematopoietic (WT to KO) locus of IFNAR expression impacted LPS-driven systemic IL-6 and TNF production (Supplementary Fig. 11). In the context of HFD feeding, either locus of IFNAR expression (hematopoietic or nonhematopoietic) equally impacted total body weight gain (Fig. 5c) and adipose tissue mass (eWAT, iWAT and pWAT) (Fig. 5d−f). Such parallels between hematopoietic and nonhematopoietic IFNAR expression largely correlated with similar obesity-associated immune cell infiltration into eWAT (e.g. increase in total CD3^+^CD4^+^ cells; decreases in CD3^+^CD8^+^, CD11c^−^B220^+^ F4/80^+^CD11b^+^TNF^+^, and F4/80^+^CD11b^+^IL-6^+^ cells) (Fig. 5g; Supplementary Table 3) and liver (e.g. increases in CD3^+^CD4^+^ and CD3^+^CD8^+^ cells; decreases in F4/80^+^CD11b^+^, percent F4/80^+^CD11b^+^TNF^+^, and percent F4/80^+^CD11b^+^IL-6^+^ cells) (Fig. 5h; Supplementary Table 4), and mirrored severity of glucose dysmetabolism (Fig. 5i) and hepatocellular damage (ALT 2- to 3-fold less) (Fig. 5j). Together these findings suggest that both nonhematopoietic and hematopoietic IFNAR expression are relevant contributors to obesity-associated inflammation and pathogenesis of obesity-associated sequelae.

**Fig. 6:**
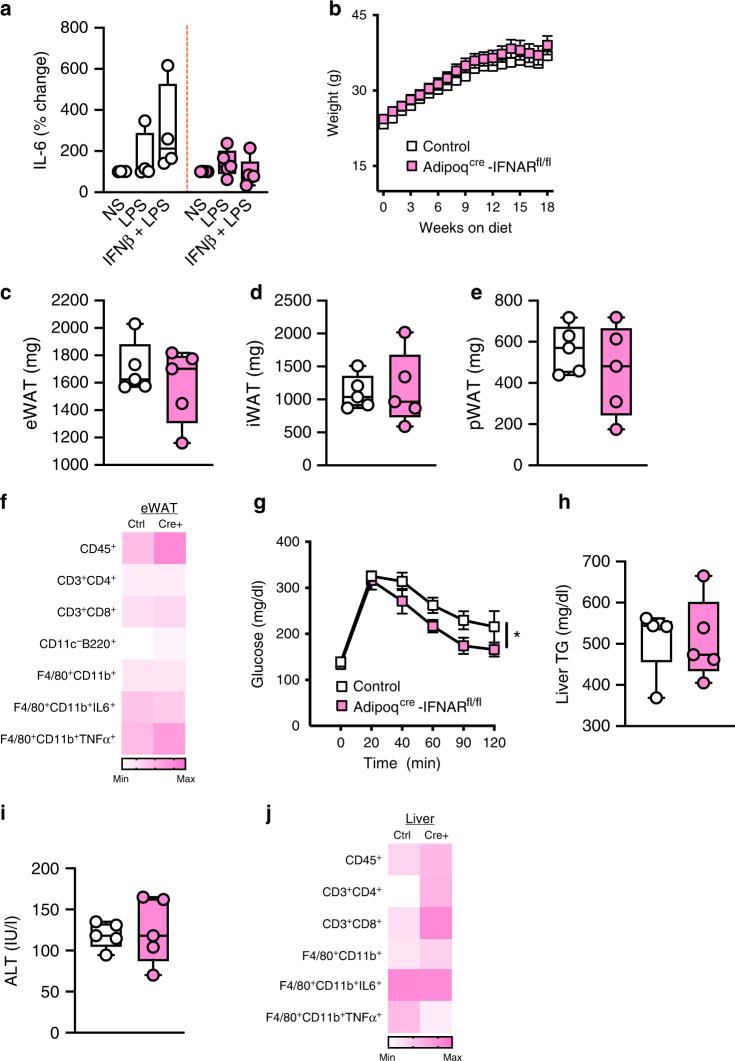
Adipocyte-intrinsic IFNAR axis is a contributor to the severity of obesity-associated sequelae. **a**−**j** Adipoq^cre^IFNAR^fl/fl^ and littermate controls (Cre^−^IFNAR^fl/fl^) were fed HFD for 18 weeks. **a** IL-6 protein in the supernatant of visceral adipose tissue-derived mature adipocytes stimulated under indicated conditions quantified by ELISA, % change to NS. **b** Total body weight. **c** eWAT, **d** iWAT, **e** pWAT weights. **f** Average eWAT immune cell infiltration, measured by flow cytometry. **g** Glucose tolerance test. **h** Liver triglycerides. **i** Systemic ALT quantified at time of harvest. **j** Average liver immune cell infiltration measured by flow cytometry. **a** Data combined from two independent experiments, *n* = 4−5/condition. **b**−**j** Representative of two independent experiments, *n* = 5/condition. **a**, **c**−**e**, **h**−**i** For box plots, the midline represents the mean, boxes represent the interquartile range and whiskers show the full range of values. **b**, **f**, **g**, **j** For line graphs and heat maps, data represent mean ± SEM. **a**, **c**−**f**, **h**−**j** Unpaired two-tailed Student’s *t* test. **b**, **g** Area under curve. *P < 0.05. Source data are provided as a Source data file.

Recent evidence indicates that FABP4^cre^-driven deletion of IFNAR expression (deletion in adipocytes, macrophages, endothelial cells, osteogenic cells, ganglion, adrenal medulla and liver^30^) may be detrimental to the pathogenesis of obesity-associated sequelae^20^. To formally delineate the contribution of adipocyte-specific IFNAR expression to the severity of obesity-associated metabolic dysfunction, we utilized Adipoq^cre^IFNAR^fl/fl^ mice^30,31^. Adipoq^cre^-driven deletion was confirmed to specifically impact IFNAR expression in adipocytes, without impacting immune cells within WAT and liver tissue (Supplementary Fig. 12). Such deletion was functional as adipocytes derived from Adipoq^cre^IFNAR^fl/fl^ mice had blunted IFNβ-driven augmentation of adipocyte inflammatory cytokine production (Fig. 6a). Adipoq^cre^ IFNAR deletion did not impact HFD-driven weight gain (Fig. 6b), adipose tissue distribution (Fig. 6c−e), or modify immune cell infiltration and macrophage inflammatory vigor in eWAT (Fig. 6f; Supplementary Table 5). Despite similar body weight, Adipoq^cre^ IFNAR deletion did impact obesity-associated glucose dysmetabolism (Fig. 6g), without modulating liver triglyceride levels (Fig. 6h) or hepatocellular damage (Fig. 6i). The lack of significant impact on hepatocellular status as a result of Adipoq^cre^ IFNAR deletion correlated with nondifferential liver immune cell infiltration including total immune cells (CD45^+^), macrophages (F4/80^+^CD11b^+^) and T cells (CD3^+^CD4^+^, CD3^+^CD8^+^) (Fig. 6j; Supplementary Table 6). Combined, these findings in adipocyte-specific IFNAR expression, via Adipoq^cre^-driven IFNAR deletion, are in partial agreement with total body IFNAR^−/−^ and nonhematopoietic IFNAR^−/−^ mice (uncoupling of body weight from severity of glucose dysmetabolism), and FABP4^cre^-driven IFNAR deletion^20^ (minimal impact on hepatocellular disease).

### Type I IFN effects on adipocytes and obesity are conserved in humans

**Fig. 7:**
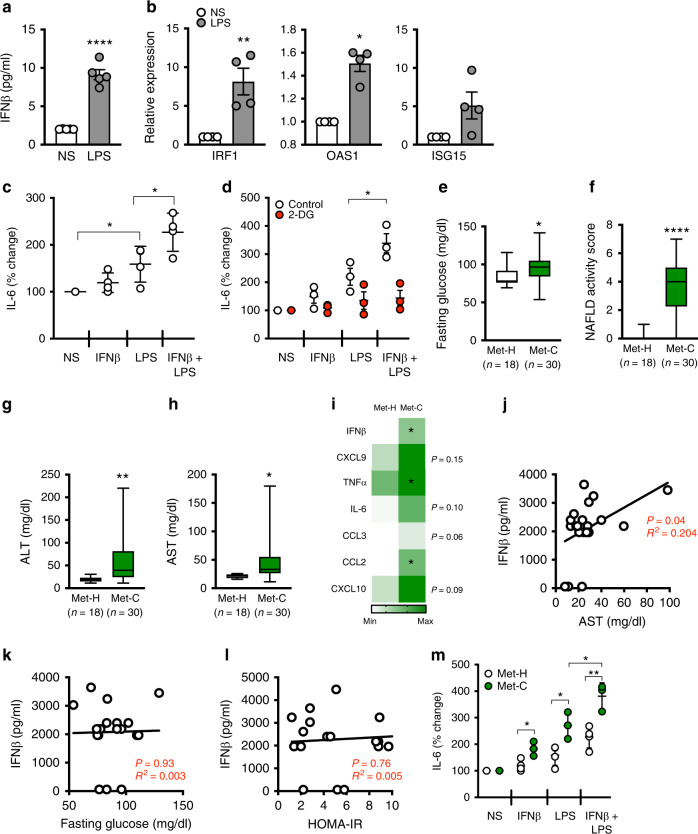
Type I IFN axis effects on adipocyte inflammatory vigor are conserved in humans. **a**−**d** Human primary adipocytes were isolated from omental adipose tissue of metabolically health obese (Met-H) individuals. Adipocytes were subsequently treated with saline (NS), human IFNβ (250 U/ml) or LPS (100 ng/ml) as indicated. **a** Quantified supernatant IFNβ protein by ELISA. **b** mRNA expression of indicated type I IFN axis genes by qPCR, relative expression to NS. **c** IL-6 protein levels quantified in supernatant by ELISA; % change over NS. **d** Quantified IL-6 protein levels in supernatant of adipocytes treated in the presence or absence of 2-DG (2 μM) by ELISA, % change over NS. **e**−**m** Persons with severe obesity were stratified into Met-H or metabolically challenged (Met-C) groups. **e** Fasting glucose. **f** NAFLD activity score. **g** ALT. **h** AST. **i** Average systemic protein levels of indicated cytokines and chemokines quantified by Luminex. **j** Systemic IFNβ levels correlated with AST. **k** Systemic IFNβ correlated with fasting glucose. **l** Systemic IFNβ correlated with HOMA-IR. **m** IL-6 protein levels quantified in adipocyte supernatant by ELISA, % change over NS. **a**−**d** Representative patients, *n* = 3−5/condition. **e**, **f** Data combined of representative patients, *n* = 18 Met-H and *n* = 30 Met-C. **i** Data combined of representative patients, *n* = 11 Met-H and *n* = 12 Met-C. **j**−**l** Data combined of representative patients, *n* = 19. **m** Representative patients, *n* = 3−4/condition. **a**, **b**, **i** For bar graphs and heat maps, data represent mean ± SEM. **c**−**h**, **m** For box plots, the midline represents the mean, boxes represent the interquartile range and whiskers show the full range of values. **a**−**i**, **m** Unpaired two-tailed Student’s *t* test. *P < 0.05, **P < 0.01, ****P < 0.0001. **j**−**l** Linear regression analysis. Source data are provided as a Source data file.

To determine whether effects of type I IFN/IFNAR axis activation are conserved from mice to humans, primary adipocytes from persons undergoing bariatric procedures were examined. *ADIPOQ* and *FABP4* mRNA expression was used to determine the efficacy of adipocyte differentiation (Supplementary Fig. 13). LPS treatment of primary human adipocytes was sufficient to augment IFNβ production and to induce the mRNA expression of the type I IFN axis including *IRF1*, *OAS1* and *ISG15* (Fig. 7a, b). In addition, similar to mouse adipocytes, IFNβ treatment significantly increased the capacity of human adipocytes to produce IL-6 in response to LPS (Fig. 7c), while treatment with 2-DG was sufficient to reverse augmented IL-6 production (Fig. 7d).

Since the type I IFN/IFNAR axis activates a multitude of inflammatory signaling hubs and modulates obesity-associated metabolic derangements in mice, we next examined whether the type I IFN signature^32^ is altered in the presence of obesity-associated metabolic sequelae. Persons with severe obesity were stratified into either metabolically healthy (Met-H) or metabolically challenged (Met-C) groups according to well-established clinical parameters including markers of glucose dysmetabolism (e.g. glucose, insulin, HOMA-IR) and hepatocellular disease (e.g. NAFLD activity score (NAS), aspartate transaminase (AST), ALT, and gamma-glutamyltransferase (GGT); Fig. 7e–h; Supplementary Table 7). Notably, the two cohorts exhibited similar BMI and systemic lipid profiles (e.g. total cholesterol, low-density lipoprotein, and high-density lipoprotein; Supplementary Table 7). Met-C individuals had heightened systemic levels of type I IFN signature cytokines^32^ including IFNβ, TNF, CCL2 and a trending increase in IL-6, CCL3, CXCL9 and CXCL10 as compared to Met-H individuals (Fig. 7i; Supplementary Table 8). Furthermore, systemic IFNβ levels were correlated with a marker of hepatocellular disease (Fig. 7j) but not fasting glucose (Fig. 7k) or HOMA-IR (Fig. 7l). Together, our data suggest that detection of the type I IFN/IFNAR axis-associated signatures may positively correlate with individuals with obesity-associated hepatocellular disease.

Given enhancement of a type I IFN signature in Met-C obese individuals, we next probed whether IFNβ responsiveness in human adipocytes is altered in the presence of obesity-associated metabolic sequelae. Augmentation of IL-6 production was even greater in adipocytes from Met-C obese individuals treated with LPS, while the combination of IFNβ + LPS-treated adipocytes led to the highest IL-6 production (Fig. 7m). Collectively, these results demonstrate that the type I IFN axis and its effects on inflammatory capacity are conserved in human adipocytes and potentially exacerbated in the setting of metabolic derangements.

## Discussion

**Fig. 8:**
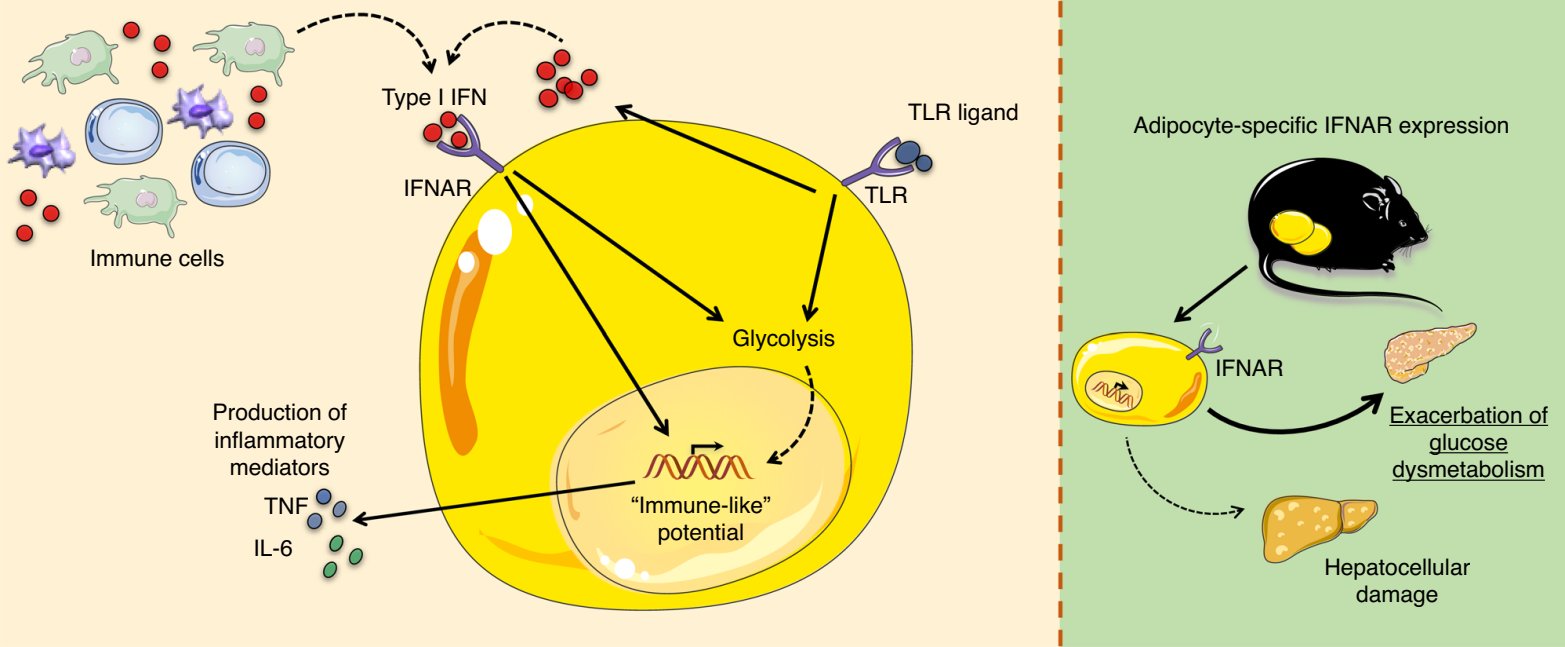
Type I IFN/IFNAR axis is a regulator of adipocyte-intrinsic inflammatory potential. A proposed model of how type I IFN/IFNAR axis unlocks adipocyte inflammatory vigor, through modulation of adipocyte core metabolism. Adipocyte-specific IFNAR expression modifies the severity of obesity-associated glucose dysmetabolism in mice with limited impact on hepatocellular damage. Source data are provided as a Source data file.

Our overall findings underscore a previously unappreciated role of type I IFNs/IFNAR axis in the regulation of adipocyte inflammatory vigor (Fig. 8). Although partial similarity in inflammatory capabilities between adipocytes and myeloid cells (e.g. proinflammatory cytokine production^6^ and expression of innate immune receptors^7^ and MHC class I and class II molecules^8,9^) have been previously alluded to, similarity of their gene expression patterns, especially in context of type I IFN sensing, has not been evaluated. The potential for the type I IFN axis to unlock a fundamental functional convergence in inflammatory gene expression patterns between adipocytes and macrophages emphasizes the complexity of WAT biology. Underpinning this complexity, our data indicate that Jak1 may be one mechanism by which the type I IFN/IFNAR axis collaborates with LPS in adipocytes. Future in-depth interrogation on the factor(s) involved in this potential synergism is clearly warranted.

Previous findings alluded that undifferentiated 3T3-L1 cells^33^ can produce IFNβ. We have expanded upon this observation by showing that primary adipocytes, both mouse and human, have the capacity to produce type I IFNs. In mice, type I IFNs are composed of 14 different IFNα subtypes and IFNω, IFNε, IFNτ, IFNκ and IFNβ. Activation of pattern-recognition receptors results in an anatomical locus/tissue/cell type-specific^34^ production of a fraction of IFNα subtypes and IFNβ. However, IFNβ is induced by LPS stimulation in myeloid cells^35^ and holds a unique ability to interact with IFNAR1 in an IFNAR2-independent manner (in addition to its ability to interact with the IFNAR1 and 2 heterodimeric complex)^36^. As adipocytes and immune cells (e.g. dendritic cells, macrophages) within the WAT can produce type I IFNs, the relevant source and subtypes of type I IFNs in the context of obesity warrants further exploration.

Our observations suggest that the type I IFN axis can alter adipocyte glycolysis. Our findings provide initial clues to how the type I IFN/IFNAR axis modifies adipocyte basal core metabolism and future comprehensive studies elucidating type I IFN/IFNAR axis impact on maximal and spare capacities in adipocytes would yield additional insights. Although lactate is a contributor to ECAR levels, other sources, including CO_2_ from the citric acid cycle^37^, likewise can contribute to acidification. Our findings suggest the possibility that IFNβ-driven modification of adipocyte core metabolism and inflammatory vigor are interlinked. IFNβ regulates glucose uptake and metabolism in cells^24,25^, and IFNβ-driven glucose metabolism is an important mechanism for the induction of antiviral responses^25^. Precise interrogation of how the type I IFN/IFNAR axis modifies the adipocyte-intrinsic metabolome to shape inflammatory vigor and immune responses would be of significant future interest. Type I IFNs are capable of altering human macrophage epigenomes to broadly reprogram their LPS-driven responses^38^ and modification of core cellular metabolism alters epigenetic and transcriptional networks associated with augmented inflammatory capacity (e.g. naïve CD4^+^ T-cell polarization, IFNγ, NF-κB signaling)^39^ in immune cells—potentially invoking another possible similarity between adipocytes and immune counterparts.

Existing literature has suggested that the type I IFN/IFNAR axis plays beneficial^19,20^ and detrimental^13–18,40,41^ roles in hepatic and metabolic dysfunction. A recent study^19^ has suggested that IFNβ overexpression in the liver, via hydrodynamic injection of a liver-targeting pLIVE-IFNβ vector, protects mice from HFD-driven weight gain. However, whether such expression promotes super-physiological levels of IFNβ in the liver and/or systemically and persistent increase in IFNβ levels drives the pathology^42^. In addition, persistent IFNβ sensing may induce exhaustion, centrally and locally, to regular IFNAR signaling^43^ akin to that known to occur in the context of TLR signaling^44^. As type I IFN/IFNAR axis is widely expressed across hematopoietic and nonhematopoietic cells, we sought to expand understanding of relevant players. Our data indicate that both hematopoietic and nonhematopoietic IFNAR expression contributes to the severity of obesity-associated metabolic sequelae (Fig. 5). FABP4^cre^-driven IFNAR deletion (gene deletion could be affected in adipocytes, macrophages, endothelial cells, osteogenic cells, ganglion, adrenal medulla and liver)^30,45^ modifies HFD-driven obesity and downstream metabolic sequelae^20^. To enhance our understanding of adipocyte-intrinsic IFNAR expression to the severity of obesity-associated metabolic disease, we employed a widely used, Adipoq^cre^-driven deletion of IFNAR^42,43^. Using this approach, we revealed that adipocyte-specific IFNAR expression contributes to obesity-associated glucose dysmetabolism (in agreement with total body IFNAR^−/−^ deletion and reciprocal BMT), but was not sufficient by itself to alter hepatocellular damage (Fig. 6). Although Adipoq^cre^ has been a widely employed line^46–48^ and off-target Cre-driven effects have not been reported, the potential of Cre-mediated toxicity is a possibility. Findings from Adipoq^cre^-driven deletion of IFNAR parallel our adipocyte transcriptome analyses, which suggest that IFNβ + LPS modified glucose tolerance, increased adipocyte glucose uptake, and altered regulation of glucose transmembrane transport (Fig. 2f). Our observations from Adipoq^cre^-driven IFNAR deletion are in partial agreement with total body IFNAR^−/−^ and nonhematopoietic IFNAR^−/−^ mice (uncoupling of body weight from severity of glucose dysmetabolism) and FABP4^cre^-driven IFNAR deletion^20^ (minimal impact on hepatocellular disease), suggesting a high level of complexity surrounding the contribution of type I IFN/IFNAR axis in obesity. Further, these data imply that additional IFNAR expressing cell types/tissues (both of hematopoietic and nonhematopoietic origin) or compensatory mechanisms likely contribute to the full phenotype spectrum observed in total body IFNAR^−/−^ mice. Notably, type I and type III IFNs share functional antiviral properties^49^ and recent evidence has emerged indicating type III IFNs in efficacy of myeloid cell responses to influenza A infection^50^. Given adipose tissue is a high energy source, microbes^51^ may harbor in adipose tissue/adipocytes. Whether the type III IFN axis is functional in adipocytes is unknown. Similarly, if type III IFNs can compensate for the lack of type I IFN signaling in the context of obesity-associated metabolic derangements is unknown and would be of pertinent future investigation.

Modulation of CD8^+^ T-cell inflammatory capacity by the type I IFN/IFNAR axis is critical for obesity-associated NAFLD pathogenesis^17^. Hepatocyte-intrinsic IFNAR expression alters hepatocyte core metabolism, subsequently impacting CD8^+^ T-cell inflammatory vigor^52^. Our data indicate that infiltration of immune cells into the liver is independent of adipocyte-specific IFNAR signaling (Fig. 6f, j). Thus, it is likely that type I IFN/IFNAR modulation of intrahepatic immune cells (e.g. CD8^+^ T cells) may be the larger governing factor to orchestrating obesity-driven NAFLD pathogenesis, while type I IFN/IFNAR activation of adipocytes underscores the severity of glucose dysmetabolism. It has also been proposed that kinetics of adipose tissue/adipocyte inflammation may drive dichotomous effects^53^. In addition to inflammation, the WAT architecture (e.g. extracellular matrix (ECM) deposition, fibrosis, etc.) promotes obesity-associated sequelae^54^. Type I IFN is a regulator of fibrosis in cutaneous graft-versus-host disease^55^. Whether the type I IFN/IFNAR axis modifies WAT architecture is unknown. Thus, future exploration of type I IFN/IFNAR control of immune cell and adipocyte inflammatory vigor and WAT architecture on the severity of obesity-associated metabolic dysfunction would provide future additional insights.

It has been posited that obesity-associated metabolic status is a consequence of a balance in “healthy vs. unhealthy” WAT states modified by inflammation and ECM^54^. Although inflammation is considered a driver of the progression to dysfunctional WAT and consequent metabolically challenged state, mechanisms underlying such processes have not been elucidated. IFNβ therapy, an approach for clinical care of multiple sclerosis patients, impairs glucose tolerance and insulin sensitivity^17^ and is associated with hepatic dysfunction^18,41^, hinting at its impact on metabolic derangement. Adult bariatric patients displayed a positive correlation between a hepatic type I IFN signature and increased TNF expression. Further, WAT isolated prior to bariatric surgery exhibited greater expression of type I IFN axis genes (e.g. *IFIT1, MX1, OAS1*)^20^, findings in line with our observations (Fig. 7). Here, we harnessed a limited pediatric cohort which represents a unique group and gives us a keen opportunity to examine the development/progression of obesity-associated metabolic derangements from a young age—mostly devoid of the accumulation of insults driving adult metabolic sequelae. Our findings indicate that systemic IFNβ levels were correlated with a marker of hepatocellular disease, but not fasting glucose or HOMA-IR. Notably, other systemic markers including TNF^56^ and IL-6^57^ that are established to correlate with HOMA-IR are similarly not associated within our cohort. However, our cohort predominantly consists of severely obese individuals without established T2D. Coupled to the limited numbers of our cohort, it is difficult to fully extrapolate strong conclusion of clinical correlates. In addition, as type I IFNs are transient mediators with rapid turnover, it is plausible that our “snapshot” is missing a critical time point in type I IFN-driven glucose dysmetabolism. Thus, future expanded enrollment of obese individuals is likely needed for additional, more in-depth exploration of the relationship between the type I IFN axis and clinical disease outcomes (e.g. T2D). Recent evidence has also begun to emerge suggesting a unique crosstalk between adipose tissue and fatty liver in both adults^58^ and adolescents^59^. Whether the activation of the type I IFN axis in adipocytes modulates the transition between healthy and unhealthy WAT remains to be determined and warrants further exploration. Additionally, definition of the similarities and differences between pediatric and adult obese patients would be of significant interest.

In sum, our report highlights a potentially undervalued role for adipocytes in the context of type I IFN-driven pathogenic diseases and suggest that greater examination of these cells could lead to expanded insights into disease pathogenesis. Thus, it is plausible that novel pharmacological intervention into the type I IFN/IFNAR axis function, in both adipocytes and immune cells, would provide alternative approaches to dampen type I IFN-driven diseases, including obesity-associated metabolic harm.

## Methods

### Mouse obesogenic diet model

All mice used were males on a C57BL/6 background (Jackson). WT, IFNAR^−/−10^, Adipoq^cre^, IFNAR^fl/fl^ mice were bred at Cincinnati Children’s Hospital Medical Center (CCHMC) in a specific pathogen-free (spf) facility maintained at 22 °C, with free access to autoclaved low-fat chow diet food (LAB Diet #5010; calories provided by carbohydrates (58%), fat (13%) and protein (29%)) and water. At 6−8 weeks of age, mice were fed either an irradiated high-fat diet (HFD; Research Diets #D12492; 60% of calories from fat) or a CD. Food was replenished on a weekly basis to avoid contamination. Total body fat, lean and water mass were determined by nuclear magnetic resonance (Whole Body Composition Analyzer; Echo MRI)^60^. Mice were fasted overnight prior to glucose metabolism testing, insulin tolerance testing, or terminal harvest. For glucose and insulin tolerance tests (ITT)^61^ mice were fasted overnight and glucose tolerance was determined by injecting mice with 10 μl of a 10% dextrose solution per gram of body weight. Glucose levels were kinetically quantified at the times indicated. For ITT, mice received 10 μl of a 0.15 U/ml solution of insulin (Novolin) per gram of body weight. Energy expenditure was measured by Phenomaster (TSE systems). Gases in the metabolic chambers were equilibrated prior to the initiation of the study and mice were acclimated for 2 days before the start of study. Data points were continuously collected for a total of 5 days. Gas exchange (O_2_ and CO_2_) was recorded every 15 min and energy expenditure (EE) was calculated according to the manufacturer guidelines. Ambient temperature (22 °C) and humidity was maintained via climate-control units in the metabolic chambers. All care was provided in accordance with the Guide for the Care and Use of Laboratory Animals. All studies were approved by the CCHMC IACUC.

### Mouse primary adipocytes

Inguinal WAT was isolated and digested (1 mg/ml Collagenase Type IV, Dispase 2, CaCl_2_)^62^. Stromal vascular fraction containing preadipocytes was cultured until confluence. Preadipocytes were subjected to initiation media (growth media (Dulbeccoʼs Modified Eagle Medium: Nutrient Mixture F-12 (DMEM:F12), Fetal Bovine Serum (FBS), Pen/Strep), rosiglitazone, dexamethasone, 3-isobutyl-1-methylxanthine, insulin) for 2 days^62^. Afterwards, cells were switched to continuation media (growth media, rosiglitazone, insulin) for 2 days, followed by differentiation media (growth media, insulin) for an additional 2 days to reach maximal differentiation. For stimulation studies, adipocytes were treated with saline (NS) or IFNβ (250 U/ml) for 3 h, followed by LPS (100 ng/ml) for 4 h. Stimulated adipocytes were utilized for downstream processes.

### Mouse epididymal WAT (eWAT)-derived mature adipocytes

eWAT was isolated from obese WT and IFNAR^−/−^ mice. After separation of mature adipocytes from stromal vascular fraction (SVF) cells using a 100 μM filter, mature adipocytes were collected and cultured in growth media (DMEM:F12, FBS, Pen/Strep) for 24 h. Mature adipocytes were treated with saline (NS) or IFNβ (250 U/ml) for 3 h, followed by LPS (100 ng/ml) for 4 h. Stimulated mature adipocytes were utilized for downstream processes.

### Human subjects

Bariatric surgery participants were recruited and informed consent obtained from the Cincinnati Children’s Hospital Medical Center (CCHMC) Pediatric Diabetes and Obesity Center. Recruitment and study protocols were approved by the institutional review board at CCHMC. Patients with alcohol abuse, viral and autoimmune hepatitis, immunosuppressive or steroid use were excluded. Liver sections were examined qualitatively using the pediatric NASH-CRN scoring system by a certified liver pathologist. The NAS is a sum of scores for steatosis, lobular inflammation and ballooning. Patients were segregated into a metabolically healthy (Met-H) or metabolically challenged (Met-C) category based on these well-established clinical parameters of hepatocellular disease (Met-H, *n* = 18; Met-C, *n* = 30; clinical phenotypes provided in Supplementary Table 7). Recruitment and study protocols were approved by the institutional review board at CCHMC.

### Human primary adipocytes

Omental WAT was collected, minced and digested with Type II collagenase (40 mg/ml in Phosphate Buffered Saline (PBS) with 2% Bovine Serum Albumin (BSA)) for 45 min at 37 °C^63^. Digested tissues were filtered and centrifuged at 250 × *g* to isolate the SVF. SVF was subjected to Ack lysis buffer. SVF was cultured (in expansion media (DMEM/F:12, 15% FBS, 1% Pen-strep)) until confluence and subjected to human adipocyte differentiation media (DMEM/F:12, 1% Pen-strep, 2 mM glutamine, 15 mM HEPES buffer solution, 10 mg/ml transferrin, 33 μM biotin, 0.5 μM insulin, 17 μM pantothenate, 0.1 μM dexamethasone, 2 nM T3, 500 μM IBMX, 1 μM ciglitazone) for 14−16d, followed by 7−10d in human adipocyte maintenance media (DMEM/F:12, 1% Pen-strep, 2 mM glutamine, 15 mM HEPES, 10 mg/ml transferrin, 33 μM biotin, 0.5 μM insulin). For stimulation studies, adipocytes were treated with saline (NS) or IFNβ (250 U/ml) for 3 h, followed by LPS (100 ng/ml) for 4 h. Stimulated adipocytes were utilized for downstream processes.

### Reciprocal bone marrow transfer and in vivo cytokine quantification

Reciprocal bone marrow transfers were generated using 8-week-old WT or IFNAR^−/−^ recipient mice^10^. 5 × 10^6^ bone marrow cells were derived from the femurs of WT or IFNAR^−/−^ mice and transferred into whole-body irradiated WT or IFNAR^−/−^ recipient mice. Peripheral blood chimerism was assessed by flow cytometric analysis 10 weeks after bone marrow reconstitution. In vivo cytokine capture assay (IVCCA) was utilized to quantify systemic IL-6 and TNF levels via biotinylated capture antibodies against IL-6 (clone MP5-32C11) and TNF (clone TN3) (both eBioscience) were given via intraperitoneal injection 3 h prior to LPS challenge and 4 h later serum cytokine levels were determined^10^.

### Epididymal WAT (eWAT) and liver immune infiltration isolation and analysis

eWAT and liver was isolated from obese WT and IFNAR^−/−^ mice. eWAT-isolated SVF cells and liver immune cells were stimulated for 4 h with PMA (50 ng/ml; Sigma-Aldrich) and Ionomycin (1 μg/ml; Calbiochem). Briefly, cells were stained with Live/dead stain (Zombie UV Dye; 1:250; Biolegend), B220 (clone RA3-6B2; 1:100; Biolegend), CD45 (clone 104; 1:500), CD11b (clone M1/70; 1:100), F4/80 (clone BM8; 1:100), Gr1 (clone RB6-8C5; 1:100), CD4 (clone GK1.5; 1:50), CD8 (clone 53-6.7; 1:100), IFNγ (clone XMG1.2; 1:100) TNF (clone MP6-XT22; 1:100), and IL-6 clone (MP5-20F3; 1:100) (all ebioscience). Data were collected using a LSR Fortessa flow cytometer (BD Biosciences) and analyzed by FlowJo software (Tree Star).

### Adipocyte cytokine quantification

Murine primary adipocytes were cultured in the presence or absence of IFNβ (250 U/ml) for 3 h and thereafter stimulated with saline, LPS (100 ng/ml), Pam2 (100 ng/ml) or Poly(I:C) (25 μg/ml) for 4 h. IL-6 was quantified by ELISA (BD biosciences) as per the manufacturer’s instructions.

### Type I IFN quantification

IFNβ levels in adipocyte culture supernatants was quantified with reference to a recombinant mouse IFNβ using an L-929 cell line transfected with an interferon-sensitive luciferase construct^10^. Luciferase activity was quantified on a SpectraMax L luminometer (Molecular Devices).

### qRT-PCR

Adipocytes were homogenized in TRIzol (Invitrogen) followed by RNA extraction, reverse transcription to cDNA (Verso cDNA Synthesis Kit; Thermo Scientific) and qPCR analysis (Light Cycler 480 II; Roche)—according to the manufacturer’s instruction^64^.

The following primer pairs were used for mouse studies: *Ifnb1* For TCCAGCTCCAAGAAAGGACG Rev TTGAAGTCCGCCCTGTAGGT; *Ifnar1* For ACACTGCCCATTGACTCTCC Rev TTGGGTGCTACCCTCAGC; *Irf9* For ACAACTGAGGCCACCATTAGAGA Rev CACCACTCGGCCACCATAG; *Oas1a* For AGCAGGTAGAGAACTCGCCA Rev CTGCATCAGGAGGTGGAGTT; *Isg15* For GTCACGGACACCAGGAAATC Rev AAGCAGCCAGCCGCAGACTG; *Il6* For TGGTACTCCAGAAGACCAGAGG Rev AACGATGATGCACTTGCAGA; *Pfk1* For CATGGGGAGAGAGGACAGA Rev AGTTCGGGAACAAGACGTTG; *Pgk1* For CAGCCTTGATCCTTTGGTTG Rev CTGACTTTGGACAAGCTGGA; *Pkm2* For GTCTGAATGAAGGCAGTCCC Rev GTCCGCTCTAGGTATCGCAG; *Ccl2* For TGTCTGGACCCATTCCTTCTTG

Rev AGATGCAGTTAACGCCCCAC; *Ccl3* For ACCATGACACTCTGCAACCAAG

Rev TTGGAGTCAGCGCAGATCTG; *Cxcl10* For CCTATGGCCCTCATTCTCAC Rev CGTCATTTTCTGCCTCATCC; *Cxcl9* For TAGGCAGGTTTGATCTCCGT Rev CGATCCACTACAAATCCCTCA; *Cxcl13* For GGCCACGGTATTCTGGAAGC Rev GGGCGTAACTTGAATCCGATCTA; *Ltb4r* For GATGCAGAAACGCACGGTC Rev GACATAGTGGCACAGGCGG; *Actb* For GGCCCAGAGCAA GAGAGGTA Rev GGTTGGCCTTAGGTTTCAGG. Arbitrary units of mRNA expression of each mouse gene were compared to *Actb*.

The following primer pairs were used for human studies: hIRF1 For CATGAGACCCTGGCTAGAGATG Rev TCCGGAACAAACAGGCATCC; hOAS1 For TGAGGTCCAGGCTCCACGCT Rev GCAGGTCGGTGCACTCCTCG; hISG15 For GAGAGGCAGCGAACTCATCT Rev CTTCAGCTCTGACACCGACA; hIL6 For CATTTGTGGTTGGGTCAGG Rev AGTGAGGAACAAGCCAGAGC; hUBIQ For CACTTGGTCCTGCGCTTGA Rev CAATTGGGAATGCAACAACTTTAT. Arbitrary units of mRNA expression of each human gene were compared Ubiquitin (human) expression.

### Lactate quantification assay

Lactate levels in adipocyte culture supernatants were quantified by colorimetric assay kit (Sigma-Aldrich) as per the manufacturer’s instructions.

### RNA sequencing and gene expression quantification

Gene expression of primary adipocytes and macrophages was determined by running 50 base pair single-end reads (~20 million reads per sample). All transcriptomic analyses were performed in StrandNGS. Following the removal of barcodes and primers, raw reads were aligned to the mm10 genome using annotations provided by UCSC with the following parameters: (1) minimum percent identify = 90; (2) maximum percent gaps = 5; (3) minimum aligned read length = 25; (4) number of matched to output per read = 1; and (5) ignore reads with more than five matches. The proprietary aligner (COBWeb) is based on the Burrow Wheeler Transform method. Aligned reads were used to compute reads per kilobase per million (RPKM) using the Expectation-Maximization algorithm for the maximum likelihood estimation of expression. Further, RPKM were thresholded at 1 and normalized using the DESeq algorithm, which computes a normalization factor (NF) for each sample. Within each sample, each transcript is divided by that transcript’s geometric mean across samples. The within-sample median of these values is that sample’s NF. To obtain normalized counts, a sample’s raw RPKM are divided by that sample’s NF. Finally, normalized per-transcript RPKM were baselined to the median of all samples. Reasonably expressed transcripts (raw RPKM > 3 in 100% of samples in at least one condition) were included for differential analysis. Differential expression was determined through two-way ANOVAs with an FDR-corrected *p* value cutoff of 0.05 and a fold change requirement of >1.5. For pathway analysis, the database at toppgene.cchmc.org was employed, which amasses ontological data from over 30 individual repositories^65^. RNA-sequencing raw data that support the findings of this study in Figs. 2, 3, Supplementary Figs. 2 and 3 can be accessed at GSE110236.

### Transcription factor binding site enrichment analysis

To identify TFs that might be regulating genes expressed in adipocytes or macrophages treated with IFNβ (250 U), LPS (100 ng/ml) or IFNβ + LPS, we utilized the RNA-sequencing analysis (above) coupled with a computational method that overlaps the genomic coordinates of a set of gene promoters with a large library of TF-genome interactions. We created the dataset library by compiling 510 mouse ChIP-seq and DNase-seq datasets from a variety of sources, including modENCODE^66^, PAZAR^67^, and the UCSC Genome Browser^68^. As input, our method takes a set of genomic regions of interest (e.g. promoters of genes whose expression changes upon stimulation), and systematically overlaps them with each ChIP-seq/DNase-seq dataset. The *observed overlap* of the input set with each dataset is calculated by counting the number of input regions it overlaps by at least one base. Next, a *P* value describing the significance of this overlap is estimated using a simulation-based procedure. A distribution of *expected overlap* values is created from 2000 iterations of randomly choosing RefSeq gene promoters with the same length as the input set (e.g. if 50 promoters of length 100 bp are used as input, then 50 randomly chosen promoters of length 100 bp will be used in each simulation). The distribution of the expected overlap values from the randomized data resembles a normal distribution and is used to generate a *Z*-score and *P* value estimating the significance of the observed number of input regions that overlap each dataset. The resulting data thus provide ranked lists of datasets (TFs, histone marks, or open chromatin), based on experimentally determined data located in the promoters of each gene set. We applied this procedure to each input gene list using three different promoter definitions: (−1000, +500), (−1000, +1000), and (−2000, +1), relative to the transcription start site. Results were similar regardless of promoter length. Additionally, we examined the underlying promoter sequences by calculating TF binding site motif enrichment scores (using the same promoter definitions). For this, we used the HOMER motif enrichment algorithm^69^, and a large library of mouse position weight matrices obtained from the CisBP database^70^. HOMER was run using the default null model.

### Cellular bioenergetics quantification

Primary adipocytes were plated at 1 × 10^4^ cells per well in a polyethylenimine precoated XF96 Cell culture microplate. Prior to bioenergetics analysis, adipocytes were treated with saline, IFNβ (250 U/ml), etomoxir (250 μM), and/or 2-DG (2 μM). An XF Analyzer (Seahorse Bioscience) was used to measure bioenergetics. Briefly, a XF96 extracellular flux assay cartridge (Seahorse Bioscience) was hydrated overnight at 37 °C according to the manufacturer’s instruction. XF assay medium supplemented with 25 mM glucose, 10 mM pyruvate and 0.3% fatty free acid BSA (pH 7.4) and incubated at 37 °C in a non-CO_2_ incubator for 1 h. For mitochondrial stress tests, Oligomycin (2 μg/ml) and carbonyl cyanide p-trifluoromethoxy-phenylhydrazone (FCCP; 1 μM) were sequentially injected and cellular oxygen consumption rate (OCR) and ECAR were quantified. For glycolysis stress test, sequential injection of glucose (2 mM), oligomycin (2 μg/ml), and 2-DG (10 mM) was performed and OCR and ECAR were quantified.

### Human adipocyte cytokine quantification

Human primary adipocytes were cultured in the presence or absence of IFNβ (250 U/ml) for 3 h and thereafter stimulated with saline, LPS (100 ng/ml), for 4 h. IL-6 (Biolegend) and IFNβ (R&D Systems) were quantified by ELISA as per the manufacturer’s instructions.

### Human systemic cytokine quantification

Human IFNβ, TNFα, IL-6, CXCL9, CCL3, and CXCL10 plasma concentrations from Met-H and Met-C patients were determined by ELISA using Milliplex^TM^ Multiplex kits (MilliporeSigma) according to the manufacturer’s protocol. Briefly, 25 μl of plasma, plated in duplicate on a 96-well black plate, was incubated with 25 μl of antibody-coated beads. Plates were washed and 25 μl of secondary antibody was incubated, followed by 25 μl of streptavidin-RPE. Sheath fluid (150 μl) was added to plates that were washed and then read using luminex technology on a Milliplex Analyzer (milliporeSigma). Data analysis was performed by the Cincinnati Children’s Medical Center Research Flow Cytometry Core.

### Statistical analysis

Statistical tests were utilized for all datasets with similar variance and analyzed by Graph. Choice of test was dependent on number of groups and whether normal distribution exists. For all normally distributed data, unpaired two-tailed Student’s *t* test was used to determine differences between groups. All data presented as means ± EM. Outlier analyses included the ROUT and Grubbs tests. Analysis was performed via GraphPad Prism Software’s. Significance is indicated as *P < 0.05, **P < 0.01, ***P < 0.001, and ****P < 0.0001. Sample sizes were determined based on preliminary data with respect to type I IFN studies in myeloid cells (e.g., myeloid cell inflammatory vigor) and obesity modeling (e.g., weight gain, immune cell infiltration and severity of obesity-associated sequelae). Our laboratory’s previous studies demonstrate that a minimal sample size of six mice in each group has a 99% power to detect a twofold difference between means with a significance level (alpha) of 0.05 (two-tailed). No animals were excluded from the analyses and none of the studies were blinded.

### Reporting summary

Further information on research design is available in the Nature Research Reporting Summary linked to this article.

## Supplementary information

Supplementary Information

Reporting Summary

## Data Availability

RNA-sequencing data supporting the findings have been deposited in GEO database and can be accessed at GSE110236. Functional enrichments of differentially expressed genes were assessed through ToppGene (https://toppgene.cchmc.org). Publicly available ChIP-seq data used in this study were obtained from mouse ENCODE^66^ (https://www.ncbi.nlm.nih.gov/geo/info/ENCODE.html#Mouse), PAZAR^67^ (http://www.cisreg.ca/), and the UCSC Genome Browser^68^ (http://genome.ucsc.edu/). Transcription factor binding site motif data were obtained from the CisBP database^70^ (http://cisbp.ccbr.utoronto.ca/). The source data underlying Figs. 1a−g, 2f, 3b, d, f, g, h, 4a−e, g−k, 5c−j, 6a−j, 7a−m and Supplementary Figs. 1a−f, 2c,  3b−d,  4a−e, 5, 6a−e, 8, 9a, b, 10b, c, 11, 12a, 13 are provided as a Source Data File.

## Source data

Source Data
