# Supplementary material for: Type I interferon sensing unlocks dormant adipocyte inflammatory potential
Source: Nat Commun. 2020 Jun 2;11:2745. doi: 10.1038/s41467-020-16571-4 (PMC7265526; doi:10.1038/s41467-020-16571-4)
Supplement: Supplementary file 1 — Supplementary Information [file 41467_2020_16571_MOESM1_ESM.pdf]

Supplementary Information

Type I Interferon Sensing Unlocks Dormant Adipocyte Inflammatory Potential

Chan et. al

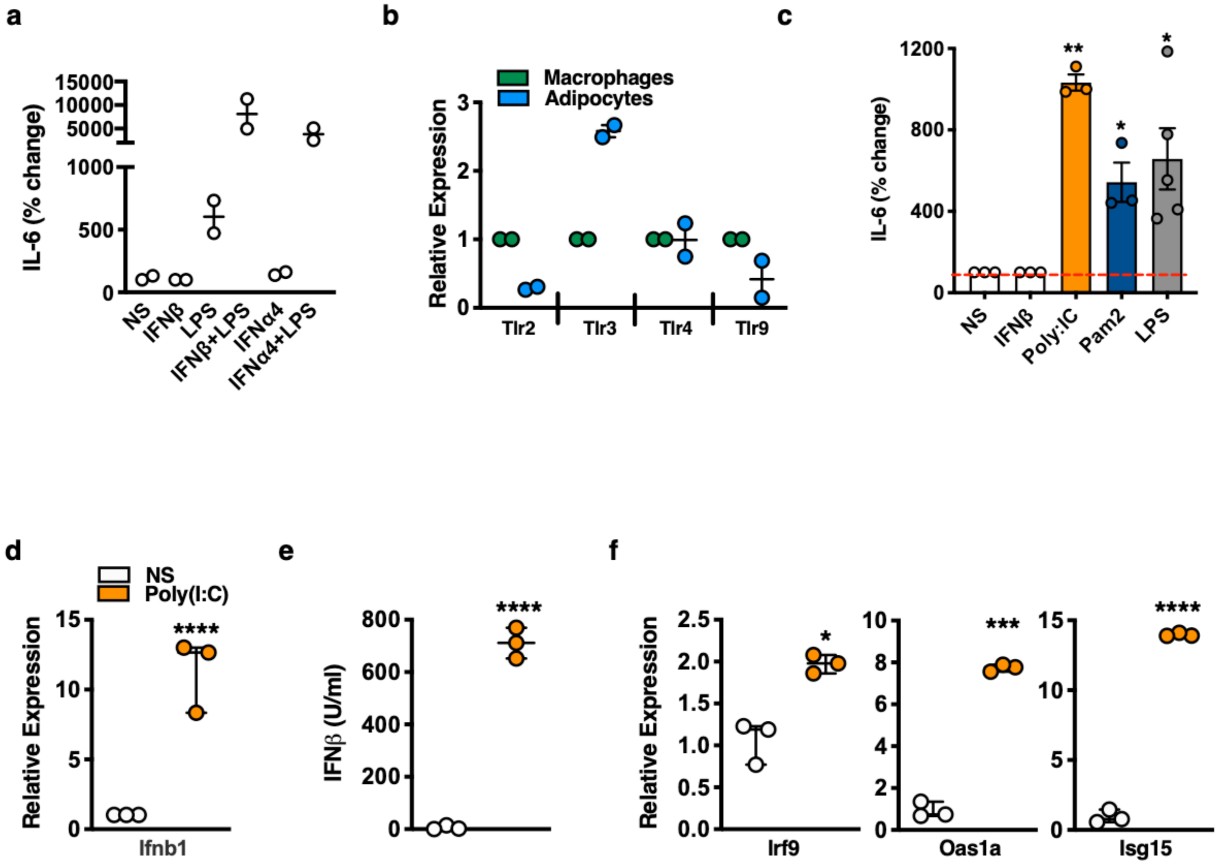

Supplementary Fig. 1. Various TLR triggers are sufficient to promote the adipocyte type I IFN axis. (**a-f**) Primary adipocytes or bone-marrow derived macrophages were isolated from CD-fed WT mice. (**a**) IL-6 protein levels in stimulated adipocytes under indicated conditions, quantified by ELISA; % change to NS. (**b**) mRNA expression by qPCR of indicated TLRs, relative expression to macrophage. (**c**) IL-6 protein levels quantified by ELISA in adipocytes treated with saline (NS), IFNβ (250 U/ml), Poly(I:C) (25 μg/ml), Pam2cys (100 ng/ml), or LPS (100 ng/ml); % change to NS. (**d-f**) Primary adipocytes isolated from WT mice were stimulated in the presence or absence of Poly(I:C) (25 μg/ml). (**d**) Ifnb1 mRNA expression by qPCR. (**e**) IFNβ protein quantified by type I IFN activity assay. (**f**) mRNA Expression of indicated type I IFN axis genes by qPCR, relative expression to NS. (**a-c**) Representative of 3 independent experiments, n = 2-5/condition. (**d-f**) Representative of 3 independent experiments, n = 3/condition. (**a-b, d-f**) For box plots, the midline represents the mean, boxes represent the interquartile range and whiskers show the full range of values. (**c**) For bar graphs, data represents mean +/- SEM. (**a-f**) Unpaired two-tailed student's t-test. *P < 0.05, **P < 0.01, ***P < 0.001, ****P < 0.0001. M denotes macrophage and A denotes adipocyte. Source data are provided as a Source data file.

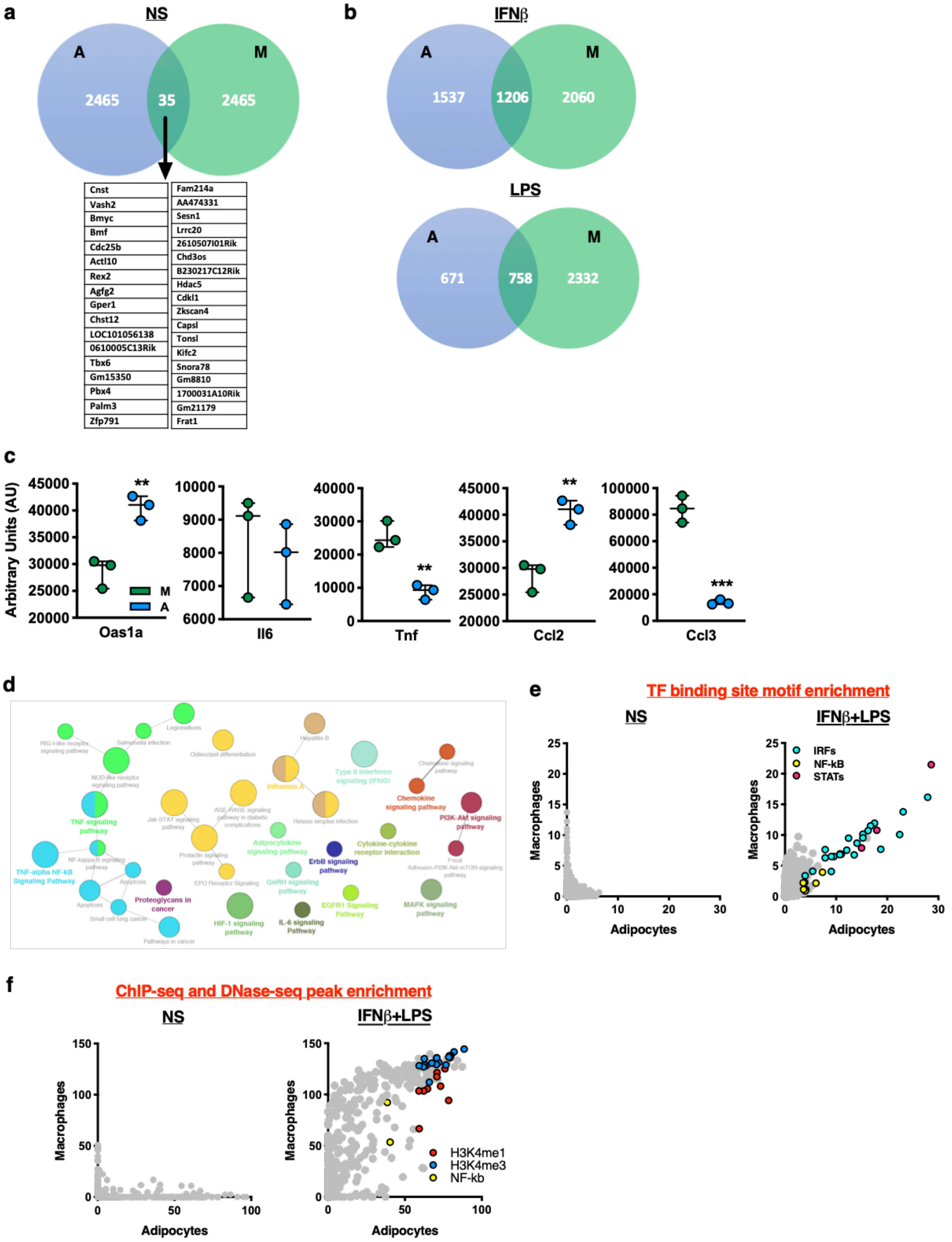

Supplementary Fig. 2. Convergence of adipocytes and macrophage gene expression patterns. Primary adipocytes and macrophages isolated from CD fed WT

mice were treated with saline (NS), IFNβ (250 U/ml) or LPS (100 ng/ml) as indicated and subjected to RNA-seq analysis. (**a-b**) Venn diagram representation of number of differentially regulated genes in adipocytes and macrophages under NS. (**c**) Validation of mRNA expression by qPCR of indicated genes, arbitrary units compared to beta-actin expression. (**d**) Common pathways in adipocytes and macrophages treated with IFNβ+LPS. (**e-f**) Complementary computational analyses of promoters of genes with elevated expression (determined by RNAseq) for TF binding site motif enrichment and ChIP-seq and DNase-seq peak enrichment. (**e**) Promoter regions of genes expressed in Adipocytes or Macrophages stimulated with saline (NS) or IFNβ+LPS (200U/100ng/ml), were inspected for over-represented predicted TF binding sites (see Methods). Each data point in the scatterplot represents one TF binding site motif, with the X- and Y-axes indicating the negative log of the p-value describing the significance of the enrichment of the motif in the given gene promoter set. Motifs for particular TF classes are colored (see inset). Results are shown for promoters defined as (-1000,+500) relative to the TSS. NS, not stimulated. (**f**) Promoter regions of genes expressed in Adipocytes or Macrophages were inspected for over-represented ChIP-seq or DNase-seq peak datasets (see Methods). Each data point in the scatterplot represents a single dataset (e.g. ChIP-seq for a particular TF or histone mark in a certain cell type). Particular types of datasets are colored (see inset). Results are shown for promoters defined as (-1000,+500) relative to the TSS. For both analyses, the X-axis shows adipocyte data sets and Y-axis displays macrophage data sets. (**a-b, d-f**) A single experiment, n = 2/condition. (**c**) Representative of 3 independent experiments, n = 3/condition. (**c**) For box plots, the midline represents the mean, boxes represent the interquartile range and whiskers show the full range of values. (**c**) Unpaired two-tailed Student's t-test. $^{**}P < 0.01$, $^{***}P < 0.001$. A denotes adipocytes. M denotes macrophages. Source data are provided as a Source data file.

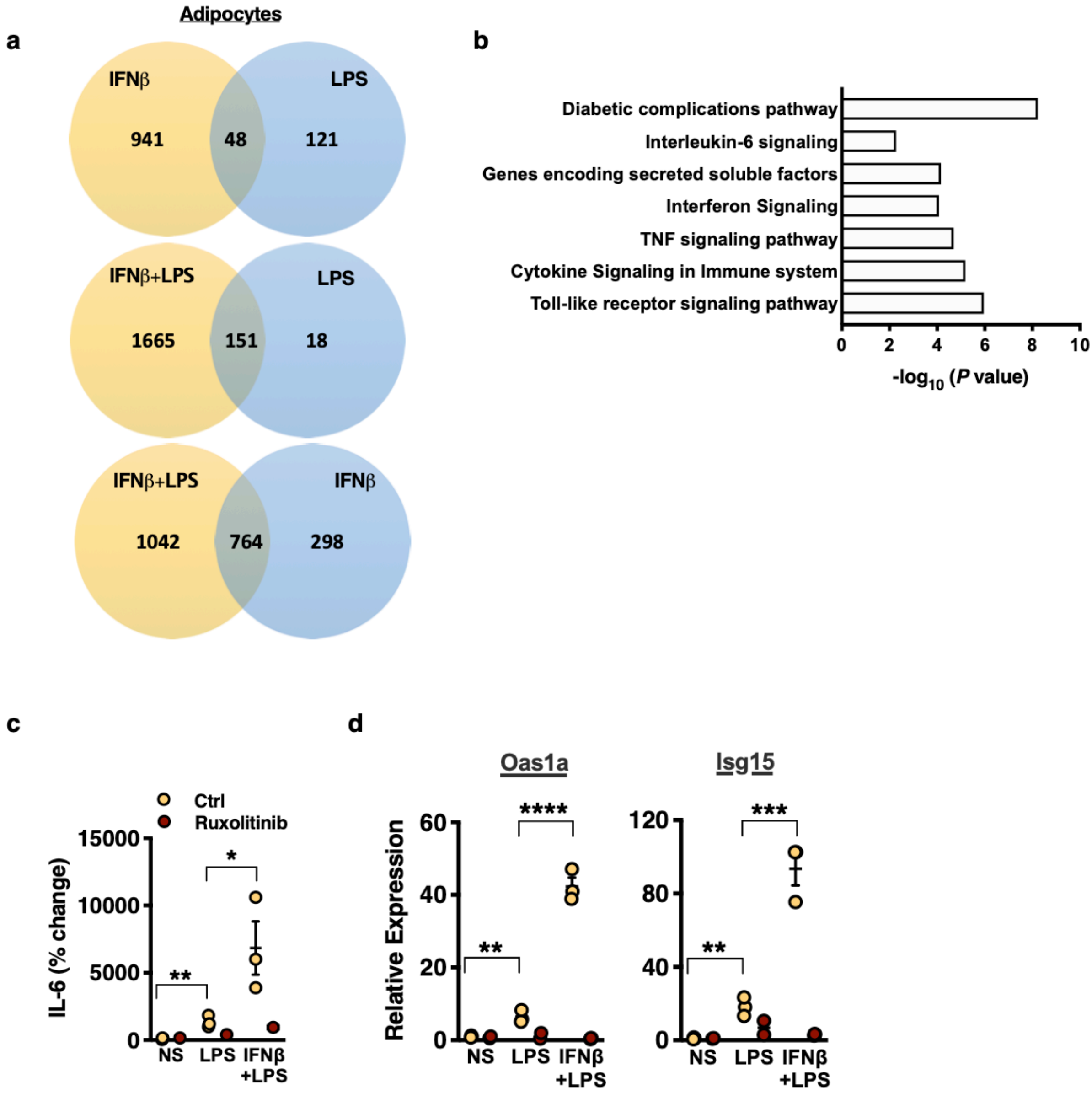

Supplementary Fig. 3. Ontologies significantly augmented by IFNβ+LPS treatment in adipocytes. (a-b) Primary adipocytes isolated from CD fed WT mice were treated with saline (NS), IFNβ (250 U/ml) and/or LPS (100 ng/ml) as indicated and subjected to RNA-seq analysis. (**a**) Venn diagram representation of number of differentially regulated genes in adipocytes under indicated treatment. (**b**) Adipocyte ontology pathways significantly augmented by IFNβ+LPS treatment (>2 fold over IFNβ or LPS alone) by RNA-seq analysis. (**c-d**) Adipocytes isolated from CD fed WT mice were treated with NS, IFNβ (250 U/ml) and/or LPS (100 ng/ml) in the presence or absence of Ruxolitnib (200 nM). (**c**) IL-6 levels in supernatant were quantified by ELISA. (**d**) mRNA expression of IFNAR axis genes as indicated, relative expression to NS. (**a-b**) A single experiment, n = 2/condition. (**c-d**) Representative of 3 independent experiments, n = 2-3/condition.

(**c-d**) For box plots, the midline represents the mean, boxes represent the interquartile range and whiskers show the full range of values. (**c-d**) Unpaired two-tailed student's t-test. *$P < 0.05$, **$P < 0.01$, *** $P < 0.001$, **** $P < 0.0001$. Source data are provided as a Source data file.

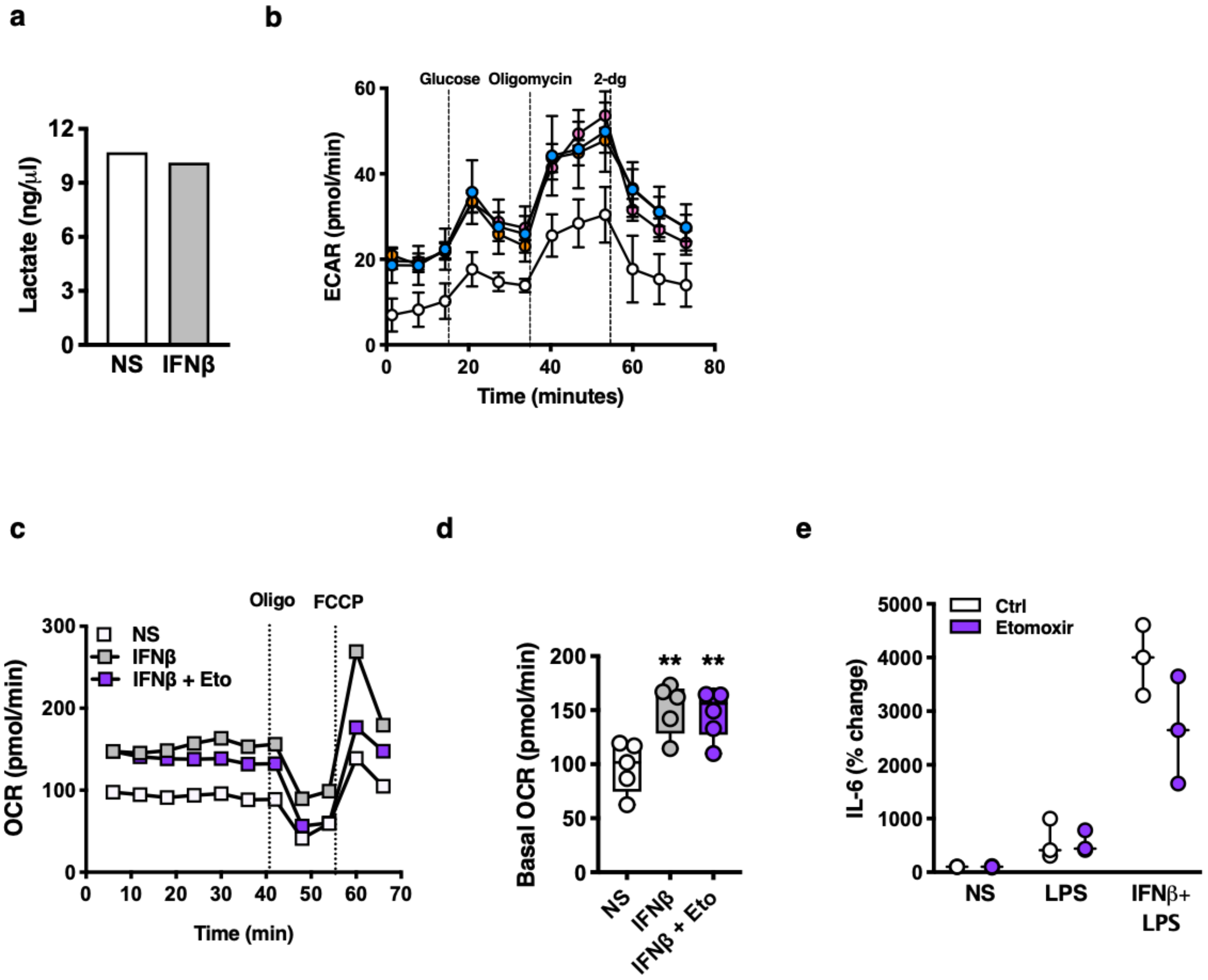

Supplementary Fig. 4. IFNβ modifies glycolysis in adipocytes. (**a**) Adipocytes treated in the presence of absence of IFNβ (250 U/ml), quantified mean lactate in adipocyte supernatants. (**b-c**) Adipocytes were treated in the presence or absence of IFNβ (250 U/ml) and/or LPS (100 ng/ml). Cellular bioenergetics of treated adipocytes was determined by Seahorse XF96 analyzer with sequential injection of glucose (2 mM), oligomycin (2 μg/ml), and 2-DG (10 mM). (**b**) ECAR. (**c-d**) Adipocytes treated in the presence or absence of IFNβ (250 U/ml) and/or Etomoxir (250 μM) and cellular bioenergetics of treated adipocytes was determined by Seahorse XF96 analyzer with sequential injection of oligomycin (2 μg/ml) and FCCP (1 mM). (**c**) OCR. (**d**) Basal OCR. (**f**) Adipocytes were treated in the presence or absence of IFNβ (250 U/ml), LPS (100 ng/ml) and/or Etomoxir (250 μM). IL-6 protein quantified in supernatants by ELISA, % change to NS. (**a**) Representative of 3 independent experiments, n = 2-3/condition. (**b-c**) Representative of 2 independent experiments, n = 4/condition. (**d-e**) Representative of 3 independent experiments, n = 5-6/condition. (**f**) Representative of 3 independent experiments, n = 3/condition. (**b-c**) For line graphs and (**d**) bar graphs, data represents mean +/- SEM. (**e**) For box plots, the midline represents the mean, boxes represent the

interquartile range and whiskers show the full range of values. (**a, e-f**) Unpaired two-tailed student's t-test. ***P* < 0.01. Source data are provided as a Source data file.

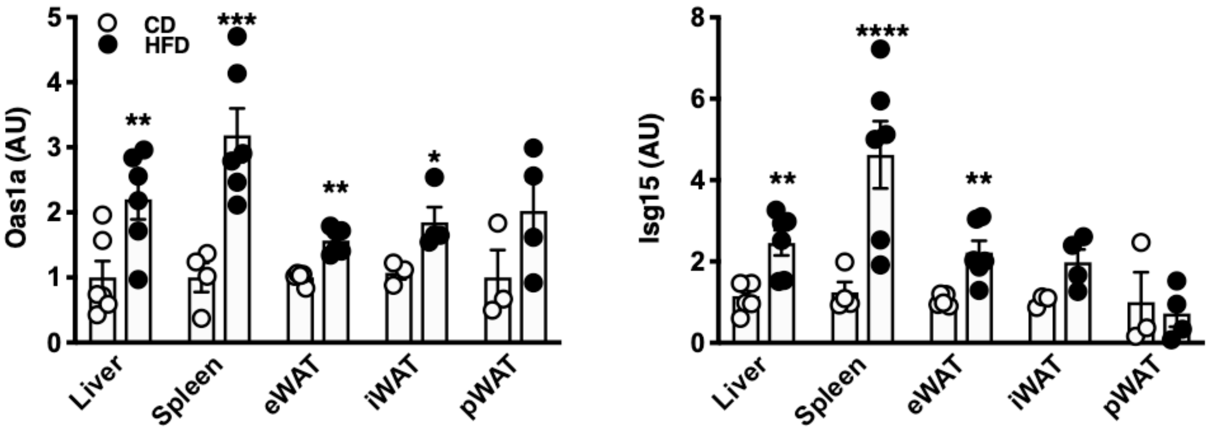

Supplementary Fig. 5. Obesity augments type I IFN signature across organs. mRNA expression by qPCR of the indicated type I IFN axis genes in liver, eWAT and spleen of WT mice fed CD or HFD for 22 weeks. Representative of 3 independent experiments, n = 3-6/condition. For bar graphs, data represents mean +/- SEM. Unpaired two-tailed student's t-test. *$P < 0.05$*, **P < 0.01, ***P < 0.001, ****P < 0.0001. Source data are provided as a Source data file.

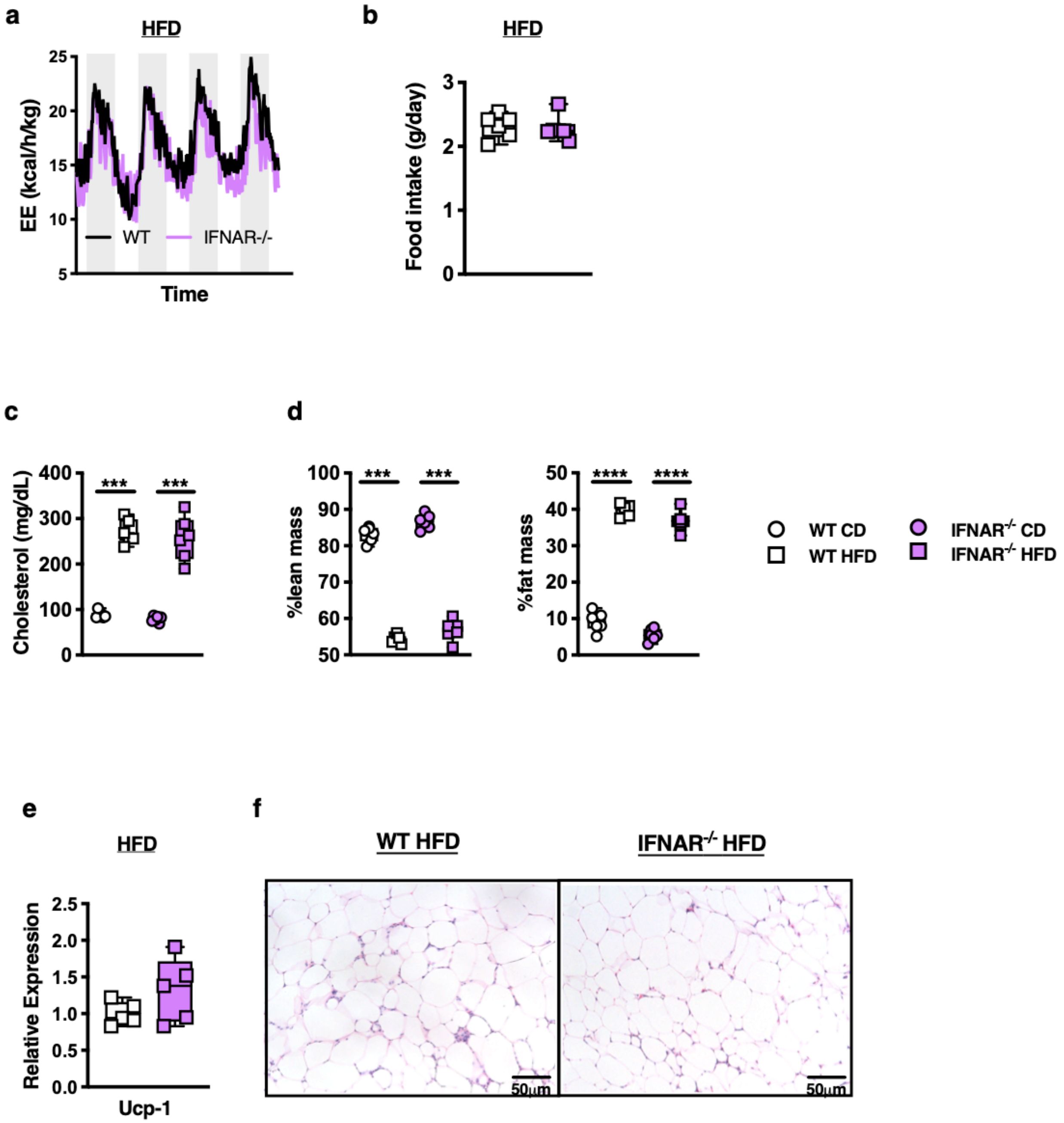

Supplementary Fig. 6. IFNAR does not modify HFD-driven obesity and adiposity. WT and IFNAR$^{-/-}$ mice were fed CD or HFD for 22 weeks. (**a**) Energy Expenditure measured by TSE systems. (**b**) Cumulative food intake between HFD-fed mice. (**c**) Systemic cholesterol. (**d**) Total body lean (%) and fat (%) mass quantified by Echo MRI. (**e**) Brown adipose tissue (BAT) Ucp-1 mRNA expression by qPCR. (**f**) eWAT H&E staining. (**a-f**) Representative of 3 independent experiments, n = 5-7/condition. (**a**) For line graph, data represents mean. (**b-e**) For box plots, the midline represents the mean, boxes represent the interquartile range and whiskers show the full range of values. (**b-e**)

Unpaired two-tailed student's t-test. ***$P < 0.001$, ****$P < 0.0001$. Source data are provided as a Source data file.

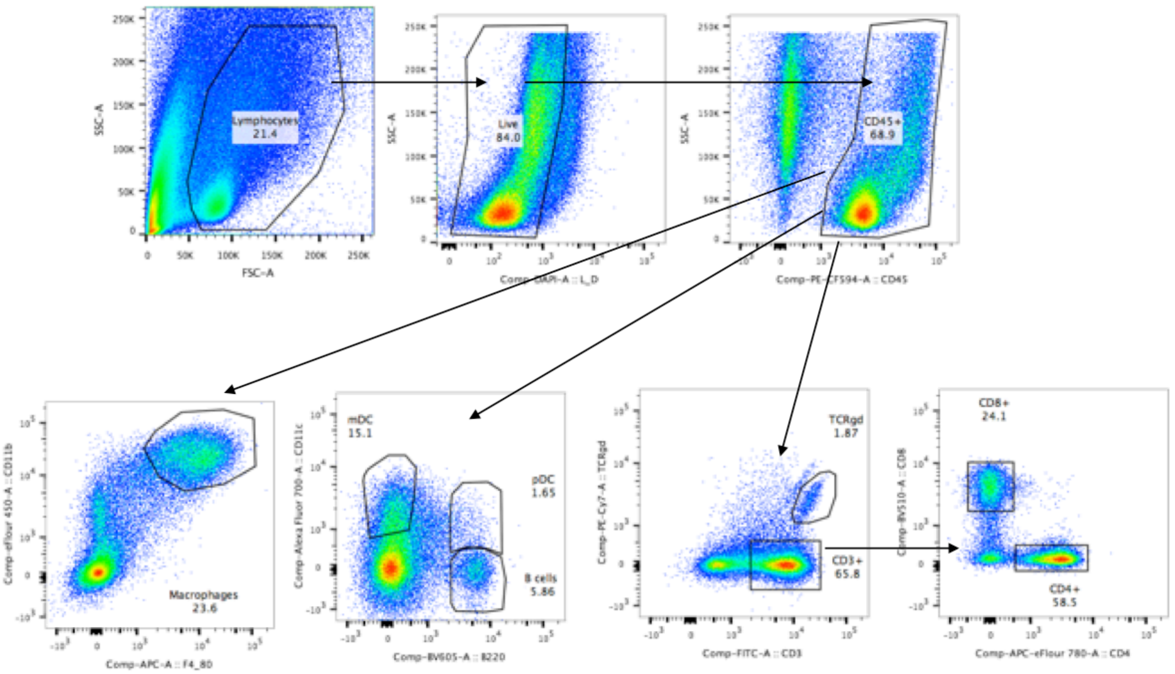

Supplementary Fig. 7. Representative gating strategy for FACS analysis of immune cell infiltration into WAT and Liver of obese IFNAR-deficient and WT mice. Additional data related to Fig. 4, a representative gating strategy for identification of $CD45^+$, $CD3^+CD4^+$, $CD3^+CD8^+$, $CD11c^-B220^+$, $F4/80^+CD11b^+$, $F4/80^+CD11b^+IL6^+$, and $F4/80^+CD11b^+TNF\alpha^+$ by flow cytometry.

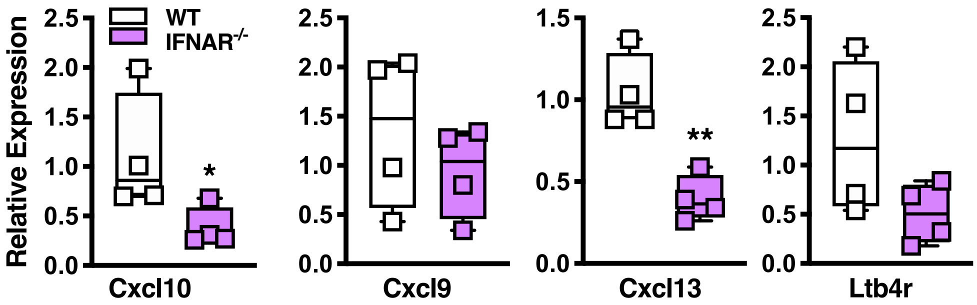

Supplementary Fig. 8. IFNAR remodels WAT inflammation.
WT and IFNAR$^{-/-}$ mice were fed HFD diet for 22 weeks. mRNA expression by qPCR of indicated chemokines in eWAT tissue. Representative of 3 independent experiments, n = 4/condition. For box plots, the midline represents the mean, boxes represent the interquartile range and whiskers show the full range of values. Unpaired two-tailed student's t-test. *P < 0.05, **P < 0.01. Source data are provided as a Source data file.

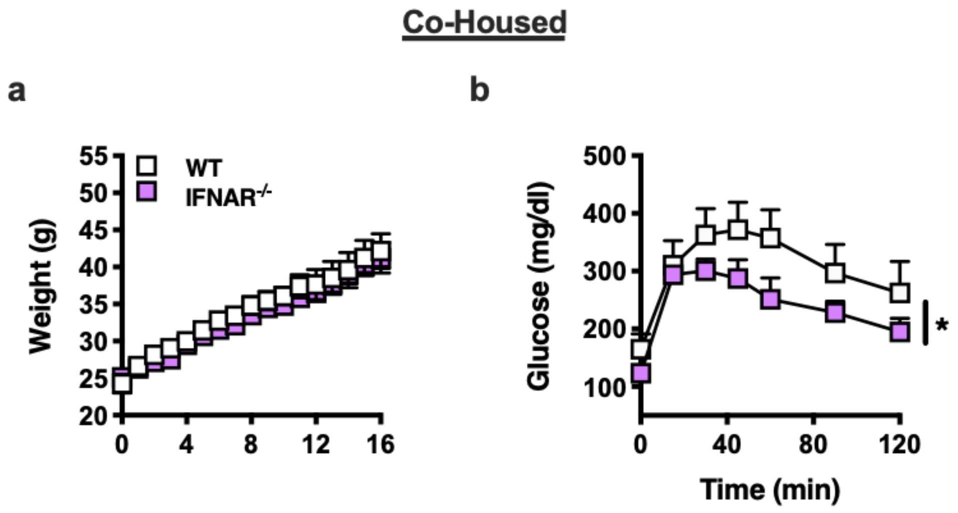

Supplementary Fig. 9. Co-housing does not impact type I IFN/IFNAR axis effects in obesity. (**a-b**) WT and IFNAR$^{-/-}$ mice were co-housed for 6 week and subsequently fed HFD for 16 weeks. (**a**) Body weight. (**b**) GTT at week 14. Representative of 3 independent experiments, n = 3/condition. (**a-b**) For line graphs, data represents mean +/- SEM. (**a-b**) Area under the curve. *p < 0.05. Source data are provided as a Source data file.

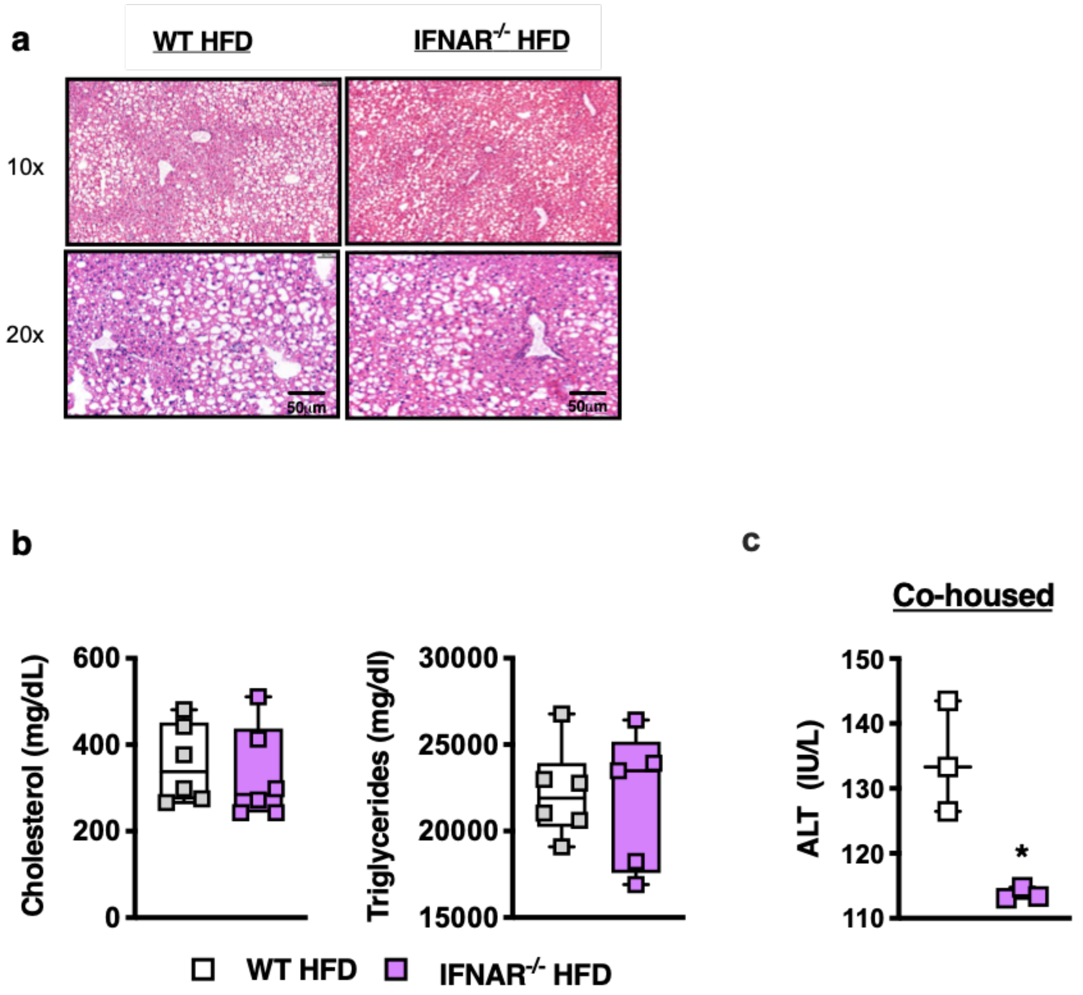

Supplementary Fig. 10. IFNAR modulates obesity-associated NAFLD pathogenesis. (**a-b**) WT and IFNAR$^{-/-}$ mice were fed an obesogenic diet for 22 weeks. (**a**) H&E stained liver tissue. (**b**) Liver cholesterol and triglyceride levels. (**c**) WT and IFNAR$^{-/-}$ mice were co-housed for 6 week and subsequently fed HFD for 16 weeks. ALT quantified at time of harvest. (**a-b**) Representative of 3 independent experiments, n = 5-6/condition. (**c**) Representative of 3 independent experiments, n = 3/condition. For bar graphs, data represents mean +/- SEM. (**b-c**) For box plots, the midline represents the mean, boxes represent the interquartile range and whiskers show the full range of values. (**b-c**) Unpaired two-tailed student's t-test. *P < 0.05. Source data are provided as a Source data file.

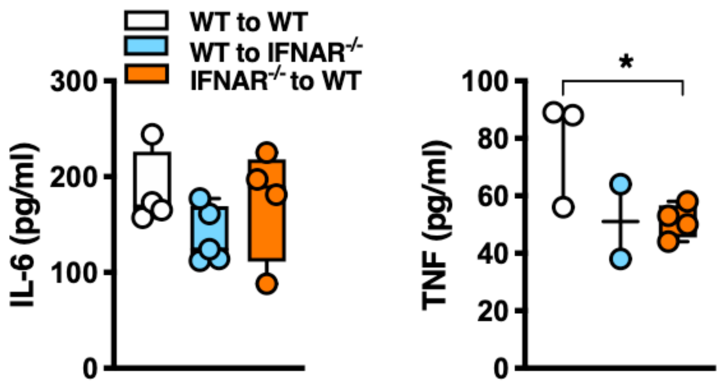

Supplementary Fig. 11. Non-hematopoietic and hematopoietic IFNAR expression comparably impact systemic proinflammatory cytokine production. Reciprocal bone marrow transfers (BMT) between WT and IFNAR$^{-/-}$ mice were performed, successful reconstitution was confirmed at d74 post-transfer by flow cytometry. LPS-driven systemic IL-6 and TNF levels in indicated lean mice quantified by *In vivo cytokine capture assay* (IVCCA). A single experiment, n = 2-5/condition. For bar graphs, data represents mean +/- SEM. For box plots, the midline represents the mean, boxes represent the interquartile range and whiskers show the full range of values. Unpaired two-tailed student's t-test. $^*P < 0.05$. Source data are provided as a Source data file.

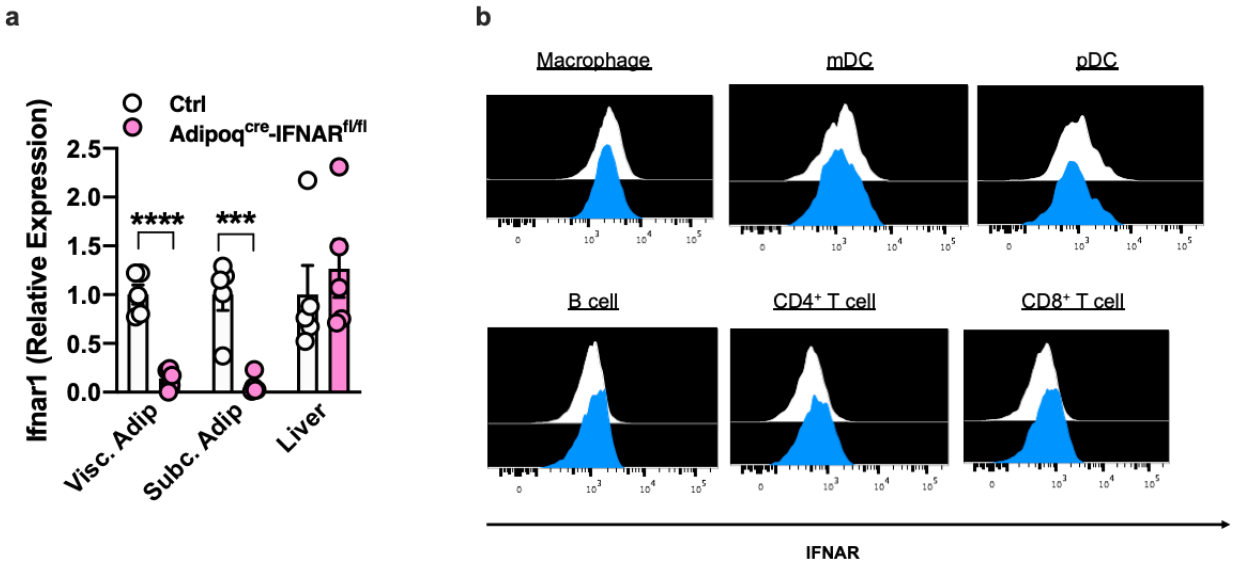

Supplementary Fig. 12. Specificity of Adipoq^{cre}IFNAR$^{fl/fl}$ deletion. Adipoq^{cre}IFNAR$^{fl/fl}$ and littermate controls were fed HFD for 18 weeks. (**a**) mRNA expression by qPCR of Ifnar1 in visceral and subcutaneous mature adipocytes and liver tissue. (**b**) Flow cytometry analysis of IFNAR expression in immune cells within eWAT of Adipoq^{cre}IFNAR$^{fl/fl}$ (blue) and littermate controls (white). (**a-b**) Data representative of 2 independent experiments, n = 5/condition. (**a**) For bar graphs, data represents mean +/- SEM. (**a**) Unpaired two-tailed student's t-test. ****P < 0.001*, *****P < 0.0001*. Source data are provided as a Source data file.

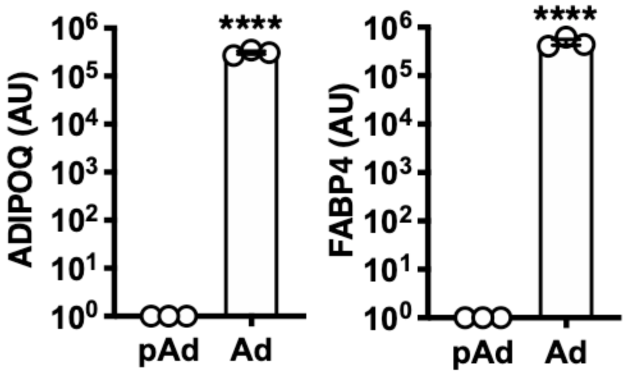

Supplementary Fig. 13. Differentiated human adipocytes display a white adipocyte gene signature. Preadipocytes (pAd) or adipocytes (Ad) were isolated from omental white adipose tissue of bariatric patients. mRNA expression by qPCR of indicated WAT signature genes, normalized arbitrary units to preadipocytes (pAd). Representative patients, n = 3/condition. For bar graphs, data represents mean +/- SEM. Unpaired two-tailed student's t-test. ****P < 0.0001. Source data are provided as a Source data file.

	WT	$IFNAR^{-/-}$	P-value
n	3	4	
$CD45^+$ ($x10^6$ mean +/- SEM)	3.46 +/- 0.89	1.13 +/- 0.12	*0.026
$CD3^+CD4^+$ ($x10^5$ mean +/- SEM)	1.37 +/- 0.3	0.38 +/- 0.07	**0.007
$CD3^+CD8^+$ ($x10^5$ mean +/- SEM)	1.99 +/- 0.51	0.58 +/- 0.09	*0.018
$CD11c^-B220^+$ ($x10^5$ mean +/- SEM)	1.54 +/- 0.35	0.26 +/- 0.04	**0.004
$F4/80^+CD11b^+$ ($x10^4$ mean +/- SEM)	1.17 +/- 0.22	1.22 +/- 0.18	0.88
$F4/80^+CD11b^+IL6^+$ (% mean +/- SEM)	15.26 +/- 2.88	8.00 +/- 1.31	*0.04
$F4/80^+CD11b^+TNF\alpha^+$ (% mean +/-SEM)	10.94 +/- 1.62	6.36 +/- 0.97	*0.03

Supplementary Table 1. eWAT immune cell infiltration in WT and $IFNAR^{-/-}$ obese mice.

	WT	$IFNAR^{-/-}$	P-value
**n**	**3**	**4**	
**$CD45^+$ ($x10^6$ mean +/- SEM)**	**1.23 +/- 0.15**	**0.54 +/- 0.15**	***0.01**
**$CD3^+CD4^+$ ($x10^5$ mean +/- SEM)**	**0.92 +/- 0.21**	**0.30 +/- 0.09**	***0.02**
**$CD3^+CD8^+$ ($x10^5$ mean +/- SEM)**	**1.78 +/- 0.58**	**0.69 +/- 0.22**	**0.09**
**$CD11c^-B220^+$ ($x10^5$ mean +/- SEM)**	**2.28 +/- 0.688**	**2.93 +/- 0.71**	**0.53**
**$F4/80^+CD11b^+IL6^+$ (% mean +/- SEM)**	**9.76 +/- 2.20**	**4.23 +/- 1.10**	***0.04**
**$F4/80^+CD11b^+TNF\alpha^+$ (% mean +/- SEM)**	**24.76 +/- 4.899**	**12.17 +/- 2.44**	***0.03**

Supplementary Table 2. Liver immune cell infiltration in WT and $IFNAR^{-/-}$ obese mice.

	$IFNAR^{-/-}$ to WT	WT to $IFNAR^{-/-}$
n	5	4
$CD45^{+}$ (x10^{6} mean +/- SEM)	3.80 +/- 1.24	5.17 +/- 0.62
$CD3^{+}CD4^{+}$ (x10^{5} mean +/- SEM)	5.83 +/- 2.12	5.67 +/- 0.62
$CD3^{+}CD8^{+}$ (x10^{5} mean +/- SEM)	0.28 +/- 0.08	0.70 +/- 0.15
$CD11c^{-}B220^{+}$ (x10^{5} mean +/- SEM)	0.88 +/- 0.36	1.35 +/- 0.44
$F4/80^{+}CD11b^{+}$ (x10^{4} mean +/- SEM)	0.75 +/- 0.28	3.24 +/- 0.50
$F4/80^{+}CD11b^{+}IL6^{+}$ (% mean +/- SEM)	1.21 +/- 0.06	1.49 +/- 0.12
$F4/80^{+}CD11b^{+}TNF\alpha^{+}$ (% mean +/- SEM)	0.93 +/- 0.21	4.54 +/- 0.80

Supplementary Table 3. eWAT immune cell infiltration in obese WT to $IFNAR^{-/-}$ reciprocal bone marrow transfer mice.

	$IFNAR^{-/-}$ to WT	WT to $IFNAR^{-/-}$
n	5	4
$CD45^+$ ($x10^6$ mean +/- SEM)	0.79 +/- 0.15	1.21 +/- 0.33
$CD3^+CD4^+$ ($x10^5$ mean +/- SEM)	1.23 +/- 0.62	0.82 +/- 0.15
$CD3^+CD8^+$ ($x10^5$ mean +/- SEM)	1.16 +/- 0.61	1.11 +/- 0.24
$F4/80^+CD11b^+$ ($x10^4$ mean +/- SEM)	1.07 +/- 0.34	0.55 +/- 0.16
$F4/80^+CD11b^+IL6^+$ (% mean +/- SEM)	3.6 +/- 0.46	1.29 +/- 0.49
$F4/80^+CD11b^+TNF\alpha^+$ (% mean +/- SEM)	9.83 +/- 1.39	0.53 +/- 0.15

Supplementary Table 4. Liver immune cell infiltration in obese WT to $IFNAR^{-/-}$ reciprocal bone marrow transfer mice.

	Ctrl	$Adipoq^{cre}IFNAR^{fl}$	P-value
n	5	5	
$CD45^+$ ($x10^5$ mean +/- SEM)	2.08 +/- 0.39	2.84 +/- 0.64	0.34
$CD3^+CD4^+$ ($x10^4$ mean +/- SEM)	3.83 +/- 0.12	3.98 +/- 0.10	0.92
$CD3^+CD8^+$ ($x10^4$ mean +/- SEM)	5.95 +/- 0.11	7.08 +/- 1.97	0.63
$CD11c^-B220^+$ ($x10^4$ mean +/- SEM)	1.17 +/- 0.19	2.78 +/- 0.8	0.08
$F4/80^+CD11b^+$ ($x10^4$ mean +/- SEM)	5.23 +/- 0.11	4.78 +/- 0.18	0.84
$F4/80^+CD11b^+IL6^+$ (% mean +/- SEM)	9.8 +/- 1.87	8.6 +/- 1.33	0.61
$F4/80^+CD11b^+TNF\alpha^+$ (% mean +/- SEM)	11.81 +/- 2.18	15.45 +/- 3.88	0.11

Supplementary Table 5. Obese $Adipoq^{cre}IFNAR^{fl/fl}$ and littermate control (Cre^- $IFNAR^{fl/fl}$) eWAT immune cell infiltration.

	Ctrl	$Adipoq^{cre}IFNAR^{fl}$	P-value
n	5	5	
$CD45^+$ (x10^5 mean +/- SEM)	4.00 +/- 1.27	6.62 +/- 0.59	0.085
$CD3^+CD4^+$ (x10^4 mean +/- SEM)	4.55 +/- 1.18	12.22 +/- 3.68	0.118
$CD3^+CD8^+$ (x10^4 mean +/- SEM)	7.8 +/- 2.42	16.32 +/- 3.73	0.116
$F4/80^+CD11b^+$ (x10^3 mean +/- SEM)	7.22 +/- 2.02	9.27 +/- 1.60	0.44
$F4/80^+CD11b^+IL6^+$ (% mean +/- SEM)	9.81 +/- 1.87	8.63 +/- 1.33	0.24
$F4/80^+CD11b^+TNF\alpha^+$ (% mean +/- SEM)	11.81 +/- 0.97	15.45 +/- 1.94	0.86

Supplementary Table 6. Obese $Adipoq^{cre}IFNAR^{fl/fl}$ and littermate control (Cre^- $IFNAR^{fl/fl}$) liver immune cell infiltration.

	Metabolically Healthy	Metabolically Challenged	P-value
n	18	30	
Age (years mean +/- SEM)	16.64 +/- 0.43	16.83 +/- 0.69	0.81
BMI (mean +/- SEM)	46.22 +/- 1.42	48.7 +/- 1.48	0.24
Glucose (mg/dL mean +/- SEM)	83.97 +/- 3.34	97.45 +/- 3.57	*0.01
Insulin (IU/mL mean +/- SEM)	37.54 +/- 7.99	53.04 +/- 11.04	0.33
HOMA-IR (mean +/- SEM)	6.75 +/- 1.39	14.11 +/- 2.82	0.07
NAFLD Activity Score (NAS; mean +/- SEM)	0.08 +/- 0.08	3.8 +/- 0.43	****<0.0001
Aspartate aminotransferase (AST; mg/dL mean +/- SEM)	20.84 +/- 0.91	39.59 +/- 5.13	*0.01
Alanine aminotransferase (ALT; mg/dL mean +/- SEM)	20.02 +/- 1.51	59.35 +/- 10.38	**0.006
Gamma-glutamyltransferase (GGT; mg/dL mean +/- SEM)	20.59 +/- 2.26	38.32 +/- 4.41	**0.005
Total Cholesterol (mg/dL mean +/- SEM)	150.6 +/- 9.06	170.2 +/- 5.93	0.059
LDL (mg/dL mean +/- SEM)	86.75 +/- 7.80	96.45 +/- 4.97	0.27
HDL (mg/dL mean +/- SEM)	31.78 +/- 2.37	30.62 +/- 1.22	0.63
Triglycerides (mg/dL mean +/- SEM)	130.6 +/- 12.93	192.6 +/- 14.08	**0.005

Supplementary Table 7. Patient cohort demographics.

	Met-H	Met-C	P-value
n	11	12	
IFNβ (pg/ml mean +/- SEM)	1794 +/- 394.6	2883 +/- 294.8	*0.035
CXCL9 (pg/ml mean +/- SEM)	2444 +/- 322.5	3924 +/- 699.5	0.15
TNFα (pg/ml mean +/- SEM)	16.83 +/- 0.99	22.7 +/- 1.50	*0.014
IL-6 (pg/ml mean +/- SEM)	7.58 +/- 2.11	17.62 +/- 4.47	0.103
CCL3 (pg/ml mean +/- SEM)	6.88 +/- 0.54	9.36 +/- 0.96	0.093
CCL2 (pg/ml mean +/- SEM)	332.9 +/- 58.09	490.3 +/- 45.49	*0.050
CXCL10 (pg/ml mean +/- SEM)	399.6 +/- 26.08	586.1 +/- 74.63	0.095

Supplementary Table 8. Met-H and Met-C systemic cytokine and chemokine levels.
