# Supplementary material for: Type I interferon sensing unlocks dormant adipocyte inflammatory potential
Source: Nat Commun. 2020 Jun 2;11:2745. doi: 10.1038/s41467-020-16571-4 (PMC7265526; doi:10.1038/s41467-020-16571-4)
Supplement: Supplementary file 2 — Reporting Summary [file 41467_2020_16571_MOESM2_ESM.pdf]

nature research

Corresponding author(s): Senad Divanovic

Reporting Summary

Nature Research wishes to improve the reproducibility of the work that we publish. This form provides structure for consistency and transparency in reporting. For further information on Nature Research policies, see Authors & Referees and the Editorial Policy Checklist.

Statistical parameters

When statistical analyses are reported, confirm that the following items are present in the relevant location (e.g. figure legend, table legend, main text, or Methods section).

n/a	Confirmed	
☐	☒	The exact sample size (n) for each experimental group/condition, given as a discrete number and unit of measurement
☐	☒	An indication of whether measurements were taken from distinct samples or whether the same sample was measured repeatedly
☐	☒	The statistical test(s) used AND whether they are one- or two-sided<br>*Only common tests should be described solely by name; describe more complex techniques in the Methods section.*
☐	☒	A description of all covariates tested
☐	☒	A description of any assumptions or corrections, such as tests of normality and adjustment for multiple comparisons
☐	☒	A full description of the statistics including central tendency (e.g. means) or other basic estimates (e.g. regression coefficient) AND variation (e.g. standard deviation) or associated estimates of uncertainty (e.g. confidence intervals)
☐	☒	For null hypothesis testing, the test statistic (e.g. F, t, r) with confidence intervals, effect sizes, degrees of freedom and P value noted<br>*Give P values as exact values whenever suitable.*
☒	☐	For Bayesian analysis, information on the choice of priors and Markov chain Monte Carlo settings
☒	☐	For hierarchical and complex designs, identification of the appropriate level for tests and full reporting of outcomes
☒	☐	Estimates of effect sizes (e.g. Cohen's d, Pearson's r), indicating how they were calculated
☐	☒	Clearly defined error bars<br>*State explicitly what error bars represent (e.g. SD, SE, CI)*

Our web collection on statistics for biologists may be useful.

Software and code

Policy information about availability of computer code

Data collection	As stated in the methods section, for RNA-sequencing analysis, all transcriptomic analyses were performed in StrandNGS aligned with the genome using UCSC. For pathway analysis, the database at toppgene.cchmc.org was employed.
Data analysis	As noted in the methods section, all statistical data analysis was performed via GraphPad Prism 8 Software. Flow cytometry was analyzed FlowJo software. GEO generated from RNA-seq analysis through TopGene. Transcription factor binding site motif enrichment was analyzed using the HOMER motif enrichment algorithm.

For manuscripts utilizing custom algorithms or software that are central to the research but not yet described in published literature, software must be made available to editors/reviewers upon request. We strongly encourage code deposition in a community repository (e.g. GitHub). See the Nature Research guidelines for submitting code & software for further information.

Data

March 2018

Policy information about availability of data

All manuscripts must include a data availability statement. This statement should provide the following information, where applicable:

- Accession codes, unique identifiers, or web links for publicly available datasets
- A list of figures that have associated raw data
- A description of any restrictions on data availability

RNASeq data for both Adipocytes and Macrophages can be found at: http://www.ncbi.nlm.nih.gov/geo/query/acc.cgi?token=otqvcueqttixngb&acc=GSE110236. The access number can be located in the methods section of the manuscript.

Field-specific reporting

Please select the best fit for your research. If you are not sure, read the appropriate sections before making your selection.

[x] Life sciences   [ ] Behavioural & social sciences

For a reference copy of the document with all sections, see nature.com/authors/policies/ReportingSummary-flat.pdf

Life sciences

Study design

All studies must disclose on these points even when the disclosure is negative.

Sample size	Under the methods section and statistical analysis subheading, adequate sample sizes were determined employing power size calculations based on the results of preliminary data.
Data exclusions	No data was excluded in this study.
Replication	All experimental findings were reproduced, with the exception of RNA-sequencing analyses and reciprocal bone marrow transfer experiment, a minimum of 2-3 times and all replication attempts were successful. Representative genes from RNA-sequencing analyses were validated and can be found in Supplementary Figure 2c.
Randomization	No randomization for experimental groups was used in this study, but animal experiments were age-matched and housed in the same box for the duration of the diet-induced obesity models.
Blinding	No blinding was used in this study given that the HFD-driven obesity models utilized in these studies leads to mice with easily distinguishable sizes.

Materials & experimental systems

Policy information about availability of materials

n/a	Involved in the study	
[x]	[ ]	Unique materials
[ ]	[x]	Antibodies
[x]	[ ]	Eukaryotic cell lines
[ ]	[x]	Research animals
[ ]	[x]	Human research participants

Antibodies

Antibodies used	As described in the methods section, antibodies used were the following: Live/dead stain (Zombie UV Dye; Biolegend; catalog 423107; lot B217753), B220 (clone RA3-6B2; Biolegend; catalog 103243),TNF (clone MP6-XT22; BDbiosciences; catalog 506333; lot B240035), IL-6 (clone MP5-20F3; BDbiosciences; catalog 561376; lot 87419) CD45 (clone 104; catalog 109845; lot B194954), CD11b (clone M1/70; catalog 12-0112-82; lot E01073-1632), F4/80 (clone BM8; catalog 17-4801-82; lot 4306323), CD4 (clone GK1.5; catalog 11-0042-85), CD8 (clone 53-6.7; catalog 48-0081-82; Lot 4299474), and IFNy (clone XMG1.2; catalog 25-7311-82; lot 4332567) [all ebioscience]
Validation	Antibodies were purchased directly from the vendors listed above and have been rigorously tested, validated, and utilized in recent publications (Moreno-Fernandez et. al 2018 JCI Insight; Giles et. al 2017 Nat. Med.; Cappelletti et. al 2017 JCI Insight).

Research animals

Policy information about studies involving animals; ARRIVE guidelines recommended for reporting animal research

Animals/animal-derived materials	As described in the methods section, male C57BL/6 background (Jackson) WT, IFNAR-/-, Adipoq-Cre, IFNAR-Flox were bred in house at Cincinnati Children's Hospital Medical Center (CCHMC) and mice aged between 6-8 weeks were utilized for studies.

Human research participants

Policy information about studies involving human research participants

Population characteristics	As described in the methods section, bariatric surgery participants were recruited from the Cincinnati Children's Hospital Medical Center (CCHMC) Pediatric Diabetes and Obesity Center. Patients with alcohol abuse, viral and autoimmune hepatitis, immunosuppressive or steroid use were not recruited. Liver sections were examined qualitatively using the pediatric NASH-CRN scoring system by a certified liver pathologist. The NAFLD activity score (NAS) is a sum of scores for steatosis, lobular inflammation and ballooning. Patients were segregated into a metabolically healthy (Met-H) or metabolically challenged (Met-C) category based on these well-established clinical parameters of hepatocellular disease (Met. H, n = 18; Met. C, n = 30; clinical phenotypes provided in Supplementary Table 1).

Method-specific reporting

n/a	Involved in the study
☒	☐ ChIP-seq
☐	☒ Flow cytometry
☒	☐ Magnetic resonance imaging

Flow Cytometry

Plots

Confirm that:

☒ The axis labels state the marker and fluorochrome used (e.g. CD4-FITC).

☒ The axis scales are clearly visible. Include numbers along axes only for bottom left plot of group (a 'group' is an analysis of identical markers).

☒ All plots are contour plots with outliers or pseudocolor plots.

☒ A numerical value for number of cells or percentage (with statistics) is provided.

Methodology

Sample preparation	As described in the methods, eWAT and liver immune cells were isolated from obese WT ,IFNAR-/-, and Adipoq-Cre/IFNAR-Flox mice as previously described (Weisberg et. al 2003 JCI; Giles et. al 2017 Nat. Med.; Moreno-Fernandez et. al 2018 JCI Insight).
Instrument	LSR Fortessa (BD biosciences)
Software	FlowJo X 10.0.7r2 was used for analysis.
Cell population abundance	Purity of immune cells was determined by expression of CD45 and gating strategy detailed below.
Gating strategy	Lymphocyte and macrophage populations were gated on a forward scatter (FSC)/side scatter (SSC) plot. Live cells were then gated using Zombie UV (Biolegend). Hematopoietic cells were further gated using CD45 to determine: CD4 T cell (CD3+CD4+), CD8 T cell (CD3+CD8+), B cells (B220+CD11c-F4/80-CD11b-), macrophages (F4/80+CD11b+). For macrophages cytokines production cells were gated using F4/80+CD11b+IL6+ and F4/80+CD11b+TNFa+. Given limited information available on the nature instructions "for authors" website regarding the need to include a specific figure exemplifying the gating strategy, if needed, such figure can be provided for this initial submission.

☒ Tick this box to confirm that a figure exemplifying the gating strategy is provided in the Supplementary Information.
